# Crosstalk Between Microglia and Müller Glia in the Age-Related Macular Degeneration: Role and Therapeutic Value of Neuroinflammation

**DOI:** 10.14336/AD.2023.0823-3

**Published:** 2024-05-07

**Authors:** Na Zhao, Xiao-Na Hao, Jie-Min Huang, Zong-Ming Song, Ye Tao

**Affiliations:** ^1^Henan Eye Institute, Henan Eye Hospital, People’s Hospital of Zhengzhou University, Zhengzhou, China.; ^2^Department of Physiology and Neurobiology, School of Basic Medical Sciences, Zhengzhou University, Zhengzhou 450001, China.

**Keywords:** microglia, neurodegeneration, retina, age-related macular degeneration

## Abstract

Age-related macular degeneration (AMD) is a progressive neurodegeneration disease that causes photoreceptor demise and vision impairments. In AMD pathogenesis, the primary death of retinal neurons always leads to the activation of resident microglia. The migration of activated microglia to the ongoing retinal lesion and their morphological transformation from branching to ameboid-like are recognized as hallmarks of AMD pathogenesis. Activated microglia send signals to Müller cells and promote them to react correspondingly to damaging stimulus. Müller cells are a type of neuroglia cells that maintain the normal function of retinal neurons, modulating innate inflammatory responses, and stabilize retinal structure. Activated Müller cells can accelerate the progression of AMD by damaging neurons and blood vessels. Therefore, the crosstalk between microglia and Müller cells plays a homeostatic role in maintaining the retinal environment, and this interaction is complicatedly modulated. In particular, the mechanism of mutual regulation between the two glia populations is complex under pathological conditions. This paper reviews recent findings on the crosstalk between microglia and Müller glia during AMD pathology process, with special emphasis on its therapeutic potentials.

## Introduction

As a peripheral component of the central nervous system (CNS), the retina is composed of multiple layers of neurons and glial cells with complicated structure. The retina senses light stimuli and converts them into electrical signals, which are subsequently sent to the occipital cortex of the brain. Retinal microglia are specialized resident immune cells that distributed in regular arrays within the inner retina [1]. They continuously monitor the neural parenchyma and peripheral tissue microenvironment, and rapidly respond to tissue damage through the dynamic processes including activation, migration, proliferation, and enhanced phagocytosis activity. In particular, they also secrete abundant inflammatory mediators and neurotrophic factors to maintain homeostasis. Microglia can be divided into two main groups based on their cell function and surface markers: M1 (pro-inflammatory) type and M2 (anti-inflammatory) type. M1 microglia secrete pro-inflammatory factors such as tumor necrosis factor-α (TNF-α), interleukin-6 (IL-6), interleukin-1β (IL-1β), and produce reactive nitrogen and oxygen free radicals [2]. The pathological insults rapidly trigger M1 microglia activation which would aggravate neuronal damage. Over-activation of M1-type microglia can produce a chronic inflammatory environment and promote the progression of AMD [3]. Conversely, M2 microglia can release a number of protective and nutritional factors that improve phagocytosis and induce immunosuppressive responses. M2 microglia use several anti-inflammatory cytokines, including interleukin-4 (IL-4), interleukin-13 (IL-13), interleukin-10 (IL-10), and transforming growth factor-β (TGF-β), to combat the pro-inflammatory response [4, 5]. They also play an important role in immune homeostasis by scavenging unwanted mannose glycoproteins. Müller cell is a type of microglial which has peripheral processes that densely infiltrate into all retinal layers. Müller cells not only play the role of scaffold and filling in the retinal structure, but also participate in the protein changes related to pathological insults. The alteration of Müller cells reactivity to injury may have cytotoxic effects on retinal neurons. Under pathophysiological conditions, Müller cells are activated to produce glial hyperplasia that damages retinal tissue and aggravates neuronal death. Possible triggers for gliosis ranging from "conserved" to "massive" gliosis will breakdown the blood-retinal barrier, resulting in an increase in the retinal and vitreous contents of growth factors, cytokines, and inflammatory factors [6].

**Figure 1.**
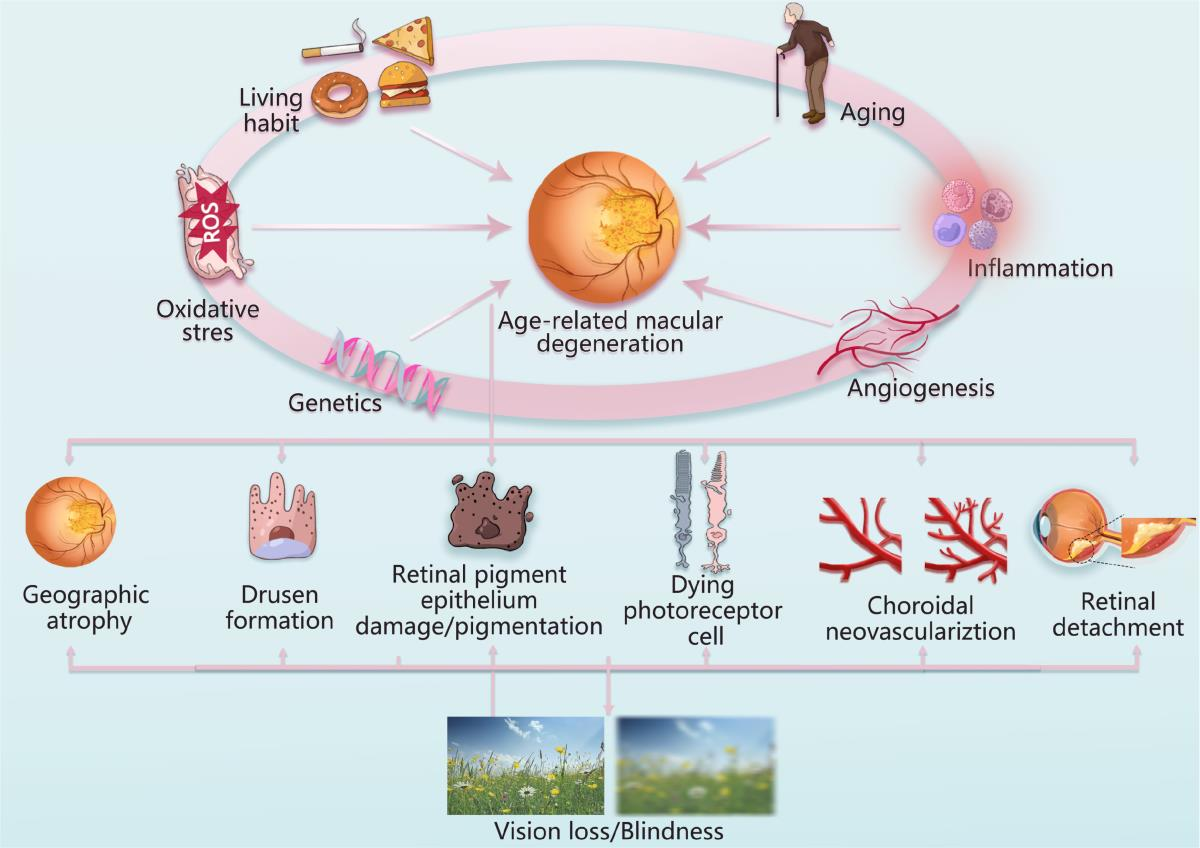
**Pathogenetic factors and pathological features of age-related macular degeneration**. Aging, inflammation, genetics, oxidative stress, angiogenesis, and Living habit are closely correlated with the development of AMD. The pathological features of AMD are geographic atrophy, drusen formation, retinal pigment epithelium damage, pigmentation, dying photoreceptor cell, choroidal neovasculariztion, retinal detachment.

AMD is a degenerative neurological disease that is characterized by progressive apoptosis of retinal neurons, yellow lipofuscin deposition, geographic retinal atrophy and loss of central vision. AMD is the main cause of irreversible blindness in elderly population, with the global prevalence of more than 288 million [7]. The exact pathogenesis of AMD remains elusive. Pathological factors such as aging, smoking, high-fat diet, and genetic factors collectively contribute to the development of AMD [8]. Notably, inflammation of retinal tissue has been found to play a central role in the disease progression. Emerging evidences show that retinal pigment epithelium (RPE) dysfunction and photoreceptor cell apoptosis would lead to chronic inflammation within retina [9]. In addition, the accumulation of cellular debris in extracellular plaques and deposits of neurons can also cause chronic local inflammatory responses, which further aggravate the retinal damage. A common morphological feature among various phenotypes of AMD is the migration of microglia into the lower lumen of the retina [10]. The activated microglia can release cytokines which would further induce recruitment of Müller cells. In this context, microglia and Müller cells, as two main types of endogenous retinal glia, interact with each other to mediated the degenerative process of AMD [11]. They can transform morphology and function through different reactions, thus determining the degree and speed of retinal damage. Therefore, it is of great significance to study the crosstalk between microglia and Müller glia in the pathology of AMD.

## Pathogenesis factors of AMD

AMD is a multifactorial disease that is characterized by slowly progressive dysfunction of the RPE, loss of photoreceptors, and progressive retinal degeneration. At present, the precise molecular mechanism underlying AMD is not fully understood. Several pathological factors have been identified as contributors to the development of AMD, including aging, inflammation, genetic predisposition, oxidative stress, angiogenesis and lifestyle habits [12] (Fig. 1).

### Aging

Age is the biggest risk factor, and almost all cases of advanced AMD occur in the elder population over 60 years old. A meta-analysis study basing on different populations found that the prevalence of AMD increased exponentially with age, especially these advanced AMD cases (odds ratio 4.2 per decade) [13]. Bruch membrane is a kind of elastic membrane with perfect contraction ability. It undergoes the processive changes associated with aging. For instance, the cholesterol-rich fat carrying egg B100-containing lipoproteins would accumulate within the Bruch membrane over time [14]. Age-related lipid deposition may represent a precursor to AMD lesions, known as "pre-blindness". These lipoprotein-like particles can stimulate inflammatory infiltration, leading to the formation of basement membrane deposits [15]. One of the main components of lipoprotein-like particles is lipofuscin. Lipofuscin is an incomplete degradation residue of the light-related vitamin A cycle in the visual cycle and accumulates in RPE cells with age. Lipofuscin is formed when RPE cells engulf the discontinuous membrane at the end of the outer segment of the photoreceptor. Long-term accumulation of lipofuscin will result in senescence of RPE, and eventually lead to age-related eye diseases like AMD. Aging causes narrowing of choroidal capillaries, decrease of blood flow area, and increase of lipofuscin deposition [16]. Aging also affects the function of white blood cells and affects the immune system of AMD patients. For instance, Fas ligand (FasL, CD95L) is a member of the tumor necrosis factor family of cytokines that contributes to modulating the neovascularization in damaged retina [17]. In AMD, the matrix metalloproteinases (MMP) activity increases with aging, and enhances the FasL cleavage on the cell membrane, resulting in limited apoptosis of inflammatory cells [18]. Additionally, a significant increase in the expression level of oxidized proteins or lipids has been detected in the retina of AMD patient, indicating that aging further causes accumulating oxidative stress that overburdens antioxidant system [19].

### Inflammation

Inflammation plays an important role in the pathophysiology of AMD. There are solid evidences showing that chronic low-level inflammation and complement activation are involved in the formation of drusen in AMD patients. Drusen contains a variety of pro-inflammatory factors, including apolipoprotein E, coagulation protein, complement components and activators [20]. Hageman et al. find that drusen is the product of local inflammatory response due to RPE damage [21]. It is considered as a hallmark of dry AMD, which can cause sever destruction of Bruch membrane, induce the growth of choroidal capillaries, lead to macular exudation and accelerate the development of AMD [22]. During aging, the malfunction of autonomic repair system may fail to restore stressed cells to a healthy state, causing them to undergo senescence. These senescent cells secrete pro-inflammatory cytokines and chemokines such as IL-6, interleukin 8 (IL-8), TNF, IL-1α, IL-1β, monocyte chemoattractant protein (MCP)-1, MCP-2, Fractalkine/ CX3C chemokine ligand 1 (CX3CL1), and granulocyte-macrophage colony-stimulating factor (GM-CSF). These mediators further stimulate neuroglia cells and activate complement system within the retina. If pathologic stress exceeds the repair capacity of resident macrophages, additional cytokines and chemokines may be released into circulation. This release can activate the systemic immune system and initiate retinal tissue remodeling [23]. Recently, Szatmari and colleagues established an in vitro AMD model by exposing human embryonic stem cell-derived RPE (hESC-RPE) cells to H_2_O_2_. They found that mature macrophages take up these dead RPE cells and produce many pro-inflammatory cytokines, thereby activating the inflammatory process [24]. These findings highlight the fact that inflammation acts as a key pathogenesis factor of AMD. Further exploration of the inflammatory molecular web will help to provide new treatment for AMD.

### Genetics

AMD is a multifactorial disease with an intricate genetic background. The International AMD Consortium conducted a large-scale study on the AMD-related genetic loci, and they identified 52 common or rare variants which are independently associated with advanced AMD at 34 genetic loci [25, 26]. It is estimated that genetic factors account for 55%-57% of the total variability in disease risk [27]. Other studies have shown that individuals with a family history have a 50% lifetime risk of developing advanced AMD, compared with 12% for those without family history [28]. Furthermore, genetic linkage analysis of AMD families has identified multiple candidate loci on most chromosomes, demonstrating the genetic heterogeneity of the disease. Fisher et al. analyzed the results of 6 published studies in the world through genome scanning integration analysis and confirmed that the gene loci most closely related to AMD susceptibility were chromosome 10q26, 1q, 2q, 3q, and 16 [29]. Complement factor H (CFH) is located on chromosome 1q, and intron variants of *CFH* gene are highly correlated with AMD [30]. Rare genetic variations in the *complement factor I (CFI)* gene have been shown to be associated with advanced AMD progression [31]. Complement factor 2 (C2) is located on chromosome 6q and has a protective effect against advanced AMD, while the incidence of AMD is higher in individuals with unmutated genes [32]. Tissue Inhibitor of *Metalloproteinase (TIMP3)* encodes a matrix TIMP3 inhibitor that is involved in the regulation of extracellular matrix (ECM) degradation and is associated with senescence and Sorsby fundus dystrophy [33]. Allikmets et al. found that the *sub-family A, member 4 (ABCA4)* gene is associated with the development of dry AMD, and it is changed in the early stage of clinical symptoms in patients [34]. In this context, the identification of genetic variants associated with AMD progression is critical to understand the background of the disease and formulate subsequent therapeutic strategy.

### Oxidative stress

Retina is particularly affected by oxidative stress due to its high metabolic rate and oxygen consumption as well as photosensitizer molecules inside the photoreceptors. Excessive production of reactive oxygen species (ROS) can cause morphological damage and functional weakening of retina proteins, lipids, and DNA [35]. When retinas are exposed to biological and abiotic stressors such as hypoxia, the balance between oxidation and antioxidant would be disturbed. In particular, the special anatomical and metabolic characteristics of the retina provide an ideal environment for ROS production. The photoreceptor outer membrane, rich in polyunsaturated fatty acids, is easily oxidized by cytotoxic chain reactions [36]. Oxidative or metabolic stress may cause damage to retinal neurons and RPE cells, inducing an adaptive response from the immune system. In AMD, the oxidative stress over burdens the autonomic adaptive response, and these stressed retinal cells may experience senescence or apoptosis. Moreover, genetic variations in oxidative stress-related genes are closely associated with AMD risk, further supporting the role of oxidative stress in AMD pathogenesis [37].

### Angiogenesis

Stress to RPE are thought to facilitate the production of angiogenic factors that drive the formation of choroidal neovascularization (CNV) in neovascular age-related macular degeneration (nAMD) [38]. Degenerative alterations of the choroidal vessels are another possible pathological cause of angiogenesis. In the Bruch membrane of nAMD patients, a major component of the accumulated lipoprotein fragments is 7-keto cholesterol. 7-keto cholesterol promotes microglia to release cytokines, such as TNF, which mediates the expression of vascular endothelial growth factor (VEGF), thereby inducing angiogenesis in retinas [39]. Normal retinal circulation requires VEGF for healthy choroidal and retinal vasculature. However, under pathological conditions, increased VEGF secretion stimulates endothelial cell proliferation and migration, promotes CNV, and damages the RPE barrier to accelerate the development of AMD [40].

### Living habits

Epidemiological studies have shown that smoking can cause oxidative damage to retinas. Chemical oxidants in cigarette deplete retinal tissue of ascorbic acid and protein sulfhydryl groups, leading to the oxidation of DNA, lipids and proteins [41]. Cytotoxic elements in cigarette also reduce plasma antioxidant level and up-regulate inflammatory cytokine level in the retinas of AMD patients [42]. Furthermore, Dietary habits, such as zinc deficiency, can sensitize RPE cells to oxidative damage [43]. A high cholesterol diet or high-sugar diet can cause pathological features of AMD, including hyperpigmentation and atrophy of RPE, lipofuscin accumulation and photoreceptor degeneration [44]. Macular pigment can absorb high-energy short-wave blue light and inhibit oxidative damage to the retina. However, these pigments cannot be synthesized by the body itself and needs to be ingested from nutritional supplements [45]. Some studies have recognized the beneficial role of diet habits in the development of AMD. The diet containing lutein and zeaxanthin have some delaying effect in AMD patients [46]. In addition, excessive exposure to Ultraviolet radiation can cause significant oxidative stress of RPE, resulting in loss of transmembrane potential and RPE apoptosis [47]. Low oxygen and high altitude also disrupt the balance between pigment epithelium-derived factors (PEDF) and VEGF, leading to retinal neovascularization and ultimately AMD [48].

## Microglia activation and AMD

Microglia are the main resident population of innate immune cells in the neural parenchyma. In the retina microglia are presented in the manner of mononuclear phagocytes, which can also be considered as macrophages of retina. Microglia exerts both protective and deleterious effects on intraretinal homeostasis. The main function of retinal microglia is to remove the cellular debris, aging neurons and degenerative synapses [49].

**Figure 2.**
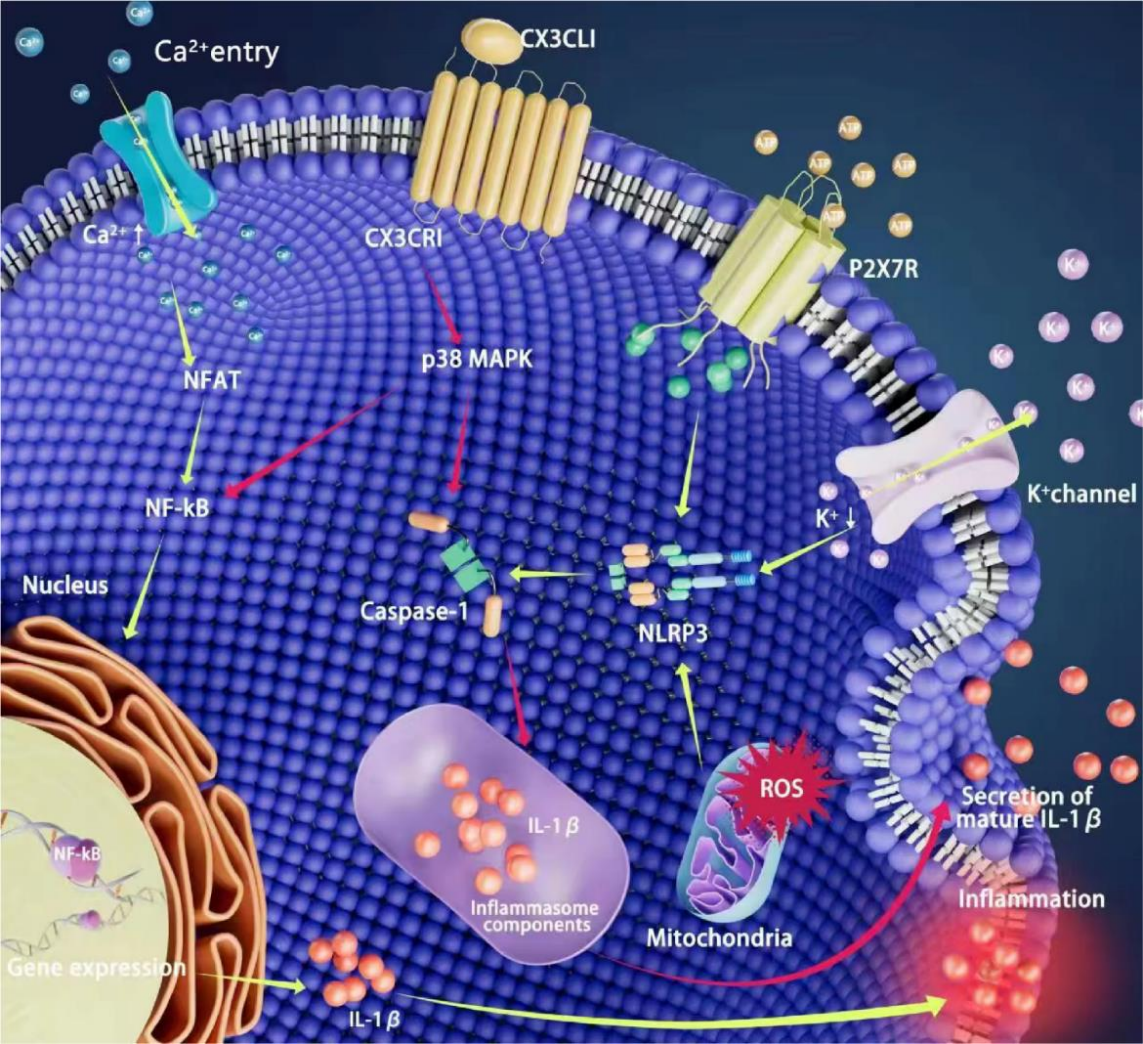
**Role of microglia in age-related macular degeneration**. Microglia can be activated in response to pathological stimuli. CX3CL1 binds to CX3CR1, the only receptor on the cell membrane, and inhibits the processing and release of mature IL-1β by caspase-1 with the active form; Extracellular ATP accelerates K^+^ efflux through ATP-gated P2X7 receptor, decreases intracellular potassium concentration, triggers activation of NLRP3 inflammasome and mediates caspase-1 activation; The activation of NF-kB promotes the transcription release of IL-1β. ROS, reactive oxygen species; NFAT, activated nuclear factor; NF-kB, The nuclear Factor Kappa-beta; NLRP3, PYD domains-containing protein 3 inflammasome; P2X7R, P2X7 receptor; IL-1β, Interleukin-1β.

Microglia has been identified as the primary immune regulator in AMD pathology. However, it is difficult to believe that microglia are the only immune cells responsible for disease progression in multifactorial diseases like AMD. In chronic conditions, recruited microglia and Müller cells activation are considered to be harmful. Activated microglia can signal to Müller cells, influencing their morphological, molecular, and functional responses. Müller cells respond to microglial activation by an upregulation of inflammatory mediators IL-1β, IL-6, and inducible nitric oxide synthase (iNOS) [50,51](Fig. 2). Müller cell responses are associated with adaptive and neuroprotective effects and do not involve expression of typical markers of gliosis. Therefore, microglia-Müller cell interactions appear to be a mode of bi-directional communications that promote the development of AMD.

### Microglia distribution and function within the retina

Under physiological conditions, microglia are distributed in regular arrays within the retina, providing continuous immune surveillance of the extracellular environment through their dynamic process movements. Ramified microglia are usually confined to the inner retina, including the nerve fiber layer, ganglion cell layer (GCL), inner plexus layer (IPL), and inner nuclear layer (INL), which are composed of synapses between retinal neurons [52]. These ramified microglia are very sensitive to detect changes in the surrounding environment, and continuously collect signals from adjacent cells by means of high-frequency extension and contraction [53]. They can respond rapidly to stimulus by altering their activation status, acquiring the ability to migrate, proliferate, and secreting inflammatory mediators. These activated microglia (Iba-1 positive microglia) exhibit synaptic degeneration and display a typical amoeboid-like appearance. Activated microglia have three states named classical activation, alternative activation and acquired inactivation. Classical activation is associated with the production of pro-inflammatory cytokines such as TNF-α, IL-1β, superoxide, nitric oxide (NO), ROS, and proteases. Microglia in this state are also known as "M1 microglia" [54]. Alternative activation is a state that is under the control of anti-inflammatory cytokines such as IL-4 and IL-13. Microglia under this state is closely associated with M2 genes that promote antiinflammation, tissue repair, and reconstruction [55]. Acquired deactivation is another state to alleviate acute inflammation, and Microglia under this state this state is induced primarily by the uptake of apoptotic cells or exposure to the anti-inflammatory cytokines such as IL-10 and TGF-β [56,57]. The interaction between pro- and anti-inflammatory cytokines ultimately leads to a chronic inflammatory response, which promotes the development of AMD.

## Molecular mechanisms of microglia involvement in AMD

### Microglia induce inflammatory response

Microglia can promote para-inflammation and increase the occurrence rate of neurodegenerative disease. Retinal microglia induce excessive inflammatory responses in retina tissue, leading to disease progression and neuron death. Microglia accumulate in the subretinal space and show molecular markers of activation, producing a local pro-inflammatory environment [58]. Microglia migration and recruitment is associated with increased chemokines and cytokines, including IL-1β, and complement activation. In particular, therapeutic approach that targeting activated microglia can reduce chemokine synthesis in the damaged retina, thereby slowing down the progression of retinal degeneration [59]. Recent study shows that these infiltrated microglia can alter RPE function through a positive feedback mechanism, which in turn leads to disruption of immune privilege associated with AMD pathogenesis. Chronic inflammation by further recruiting and activating microglia would facilities the neointimal growth toward the retina [60]. the activated microglia promote retinal angiogenesis in hypoxia stress through neurovascular coupling and guide neovascularization to avascular areas (e.g., the outer nuclear layer and macula lutea) [61].

The nuclear Factor Kappa-beta (NF-kB) is associated with increased cell survival, but in AMD it leads to microglia activation and neuronal damage through the production of neurotoxic and pro-inflammatory cytokines [62]. Isabella Palazzo et al. found that NF-kB signaling in the damaged retina was expressed in microglia only when reactive microglia were present. On the other hand, Inhibition of the NF-kB signaling pathway will reduce microglia recruitment and increase neuronal survival [63]. These findings highlight the possibility that strategies to inhibit microglia activation and inflammatory response may provide a promising therapeutic approach against AMD. Wu J et al. find that inhibiting phosphorylated p38 MAPK can effectively block the release of inflammatory factors from these activated microglia [64]. A vivo study shows that Indole-3-carbinol (I3C) effectively prevents the accumulation of amoeba microglia in the inferior lumen of the retina, thus reducing the degree of retinal damage in a light-induced mouse model [65]. Intraperitoneal injection of the selective 18KDa translocator protein (TSPO) ligand XBD173 (AC-5216, emapunil) can reduce the expression of pro-inflammatory genes such as CCL2 and IL-6 and retain more branched non-reactive microglia in the phototoxicity damaged retina. Additionally, a vivo study also shows that XBD173 treatment almost completely blocks light-induced retinal degeneration [66].

### Microglia induce Oxidative stress

Prolonged oxidative stress and constant light exposure induce chronic inflammation in the retina. Oxidative stress is associated with elevated intracellular ROS that generated mainly by electrons in the in the transport chain of hydrogen peroxide, superoxide and hydroxyl radicals [67]. ROS can enhance the expression of pro-inflammatory genes and promote the release of inflammatory cytokines from microglia. These pro-inflammatory factors further exacerbate oxidative stress by creating an amplifying loop cycle that leads to progression of AMD. Steven S et al. find that knockout of the *superoxide dismutase (SOD)-1* gene alone led to increased oxidative stress in retina, and the accumulation of microglia in the subretinal space [68]. During diverse neuropathological conditions in the retina, there is a robust increase in TSPO expression that colocalizes predominantly with activated microglia and increased intracellular ROS expression [69]. Accordingly, microglia-mediated oxidative stress plays an essential role in the pathophysiological process of RPE degeneration onset and progression.

### Microglia induce metabolic abnormalities

In physiological condition, microglia express a large number of receptors for neurotransmitters that allow them to continuously monitor and respond to neuronal activity. When the retina is damaged, microglia are activated and release various bioactive molecules to affect the microenvironment of local retina. RPE cells under the influence of activated microglia may lose cellular integrity and intercellular contact. These affected RPE cells will proliferate in an unregulated manner, losing their original uniformly spaced monolayer structure and forming irregular cell aggregates [70]. Microglia activation can induce the progressive accumulation of lipofuscin, melanolipofuscin, and melanosome granules in RPE cells, thereby disturbing the normal metabolic activities of retinas. High melanolipofuscin content within foveal RPE cell bodies and abundant lipofuscin at the perifovea suggest metabolic abnormalities, plausibly related to the population of overlying photoreceptors [71]. Retina endothelial cells secrete glycolytic metabolites, in particular lactate, that trigger changes in local microglia metabolism to favor a hyper glycolytic state. These microglia express M1 markers, and produce both proinflammatory and proangiogenic cytokines, which ultimately resulting in pathological neovascularization [72].

### Microglia induce neurotoxicity

Microglia overactivation and dysregulation might result in disastrous and progressive neurotoxic consequences. Chronically activated microglia are engaged in the phagocytosis of rod debris and exacerbate photoreceptors death by secretion of neurotoxic factors [73]. In particular, the cone photoreceptors are also highly vulnerable to the neurotoxic M1 cytokines released by microglia [74]. Under normal conditions, many growth factors and neurotrophic factors are essential for the survival of retinal neurons [75]. As the retinal degeneration starts, the activated microglia invade the degenerated photoreceptor layer and reduce the expressions of neurotrophic factors including nerve growth factor (NGF), ciliary neurotrophic factor (CNTF), and glial cell line-derived neurotrophic factor (GDNF) [76]. Oxidative stress in degenerative retinas impairs the processing of pro-NGF to mature NGF. Moreover, pro-NGF induces the production of TNF-α and activates the p38 MAPK-dependent pro-apoptotic pathway in the retina [77]. CNTF is an extracellular signaling protein mainly produced by Müller cells. CNTF initiates it signaling by interacting with its receptors which are expressed in the RPE and photoreceptors [78]. The deficiency of CNTF led to the up-regulation of visual cycle enzymes, increased 11-cis-retinal regeneration rate, and increased light sensitivity of rods and cones. These neurotoxic effects may contribute to the elevated levels of oxidative stress in the photoreceptors [79]. Additionally, the neurotoxic pro-inflammatory cytokines impair the proximal nerve stump, leading to extensive neuronal loss and axonal degeneration. Therefore, neurotoxicity induced by microglia activation may be one of the factors promoting the development of AMD.

### Interplay between microglia and RPE

RPE displays several activities necessary for retinal homeostasis, including the transport of nutrients to photoreceptors and the removal of their metabolic wastes. In AMD pathology, RPE cells play a key role in modulating the activation and infiltration of innate immune cells including microglia, neutrophils, and monocytes [80]. Excessive oxidative stress altered protein assembling, dysfunction of mitochondrial form an internal feedback loop that leads to RPE failure, and allows the accumulation of misfolded proteins and abnormal lipids, resulting in the formation of drusen-like deposits. Drusen are immunologically active deposits containing oxidative lipids, lipofuscin, complement, and other immune activating components that develop as the consequence of RPE stress. Drusen can trigger chronic inflammation in the subretinal space, in which microglia are also involved [67]. The accumulation of subretinal microglia contribute to multiple features of AMD histopathology, including localized RPE structural changes and CNV formation [81]. Subretinal space is located between the apical surface of the RPE and the outer segment of the photoreceptors. It is a particularly interesting site for studying the relationship between microglia activation and RPE injury. After translocating to the sub-retinal space, the reactive microglia attain a reactive phenotype as M1 and M2. They prominently alter the cytoskeletal structure and inflammatory gene expressions in RPE cells [82]. Activated microglia induce RPE alterations that result in an elevated chemoattractant and proangiogenic environment, which increases the recruitment and activation of immune cells and fosters the growth of neovascular vessels in the retinas of wet AMD [73]. A clinical study using donated eyes from AMD patients found that microglia accumulate in the subretinal space with a rather amoeboid, activated morphology, and display engulfed melanin or spontaneous fluorescent particles [83]. These amoeboid like microglia can affect the RPE-Bruch’s membrane, and cause pigmentation abnormalities [84]. The interaction between microglia and RPE cells causes the disruption of the outer blood retinal barrier (BRB) with the amplified recruitment of microglial cells by RPE-derived IL-6 [85].

## Müller cells homeostasis and AMD

Müller cell is the largest type of glial cell in vertebrate retina-which account for 90% of all retinal glial cells. They span the entire retina and maintain the structural stability of highly organized retinal layers [86]. Müller cells are capable of secreting neurotrophic factors, glutamic acids, and adenosine which are essential for the survival of retinal neurons. Müller cells embed themselves into neurons and serve as deformable substrates for neurite growth and branching [87]. Müller cells connected to adjacent cells through gap junctions to form a small communication web [88]. Müller cells are highly resistant to pathogenic stimuli such as ischemia, hypoxia and hypoglycemia. As the main antigen presenting cells, they processing antigens into immunogenic forms and main the normal anti-pathogenesis activities of retina cells [6]. When the retina is confronted with pathologic insults, Müller cells respond rapidly to stress stimulus from the retinal microenvironment. They produce a high level of filamentous vimentin, with overproduction of glial fibrillary acidic protein (GFAP) that is usually found in the foot end of Müller cells [89]. This process is known as gliosis and can affect the function of neurons as well as blood vessels.

In classic theory, Müller cells mediated reactive responses have both cytoprotective and cytotoxic effects on neurons. Especially in the early stages after retinal damage, Müller cell gliosis is conservative and neuroprotective [90]. Subsequently, the aggravation of damage can lead to an intensified Müller cell response, called "massive" or proliferation, in which gliosis is harmful to retinal tissue and exacerbates neuronal death. The breakdown of the BRB and increased levels of inflammatory factors in the retina and vitreous body may be the trigger that promotes gliosis to change from "conservative" to "massive" [91]. Recent study show that increased permeability of BRB can affect the gliosis of Müller cells [92]. Once the BRB is ruptured by laser damage in rats, the extravasation of plasma proteins and immunoglobulin-G may further activate the gliosis response of Müller cells [93]. In the later stages AMD, Müller cells overactivation will increase the susceptibility of neuronal ultimately lead to the development of neurodegeneration. Activated Müller cells promote the synthesis and secretion of TNF-α, interleukin, interferon, intercellular adhesion molecule-1, and NO, which are toxic to retinal neurons [94]. The unique intermediate fibers in Müller Cell and the focal upregulation of GFAP expression may associated with areas of drusen formation in AMD patients [95]. Drusen accumulates with age. Drusen are extracellular deposits composed of cell debris, lipoprotein, and amyloid deposits on the Bruch membrane below the RPE [96]. Impairment of RPE cell-Bruch membrane-choroid complex is thought to be closely related to the formation of drusen. Susceptibility genes, complement activation and cholesterol clearance system disturbances are also involved in the formation of drusen.

Massive gliosis can destroy neurons-vasculature complex and inhibit tissue repair. In some of the more severe forms of reactive gliosis, Müller cells lose their functionality and form fibrotic scars that are inhibitory to neuronal regeneration. glial scar formation at the outer edge of the neuroretina can impede the regrowth of photoreceptor outer segments [97]. In some of AMD cases at late stages, the glial scar become contractile and lead to folds or deformations in the retina causing visual distortions [98]. In addition, reactive gliosis inhibits axon regeneration, and induces secondary damage to nearby neurons and glial cells. Some of the factors released by activated Müller cells such as the VEGF may exacerbate disease progression by inducing vascular leakage and neovascularization [99]. Furthermore, the dislocation of an astroglia potassium channel (Kir4.1) on Müller cell membrane reduces the K^+^ conductance of cell membrane, leading to a serious loss of the functions involved in normal neuron-glial cell interaction [100].

## Crosstalk between Microglia and Müller cells

**Figure 3.**
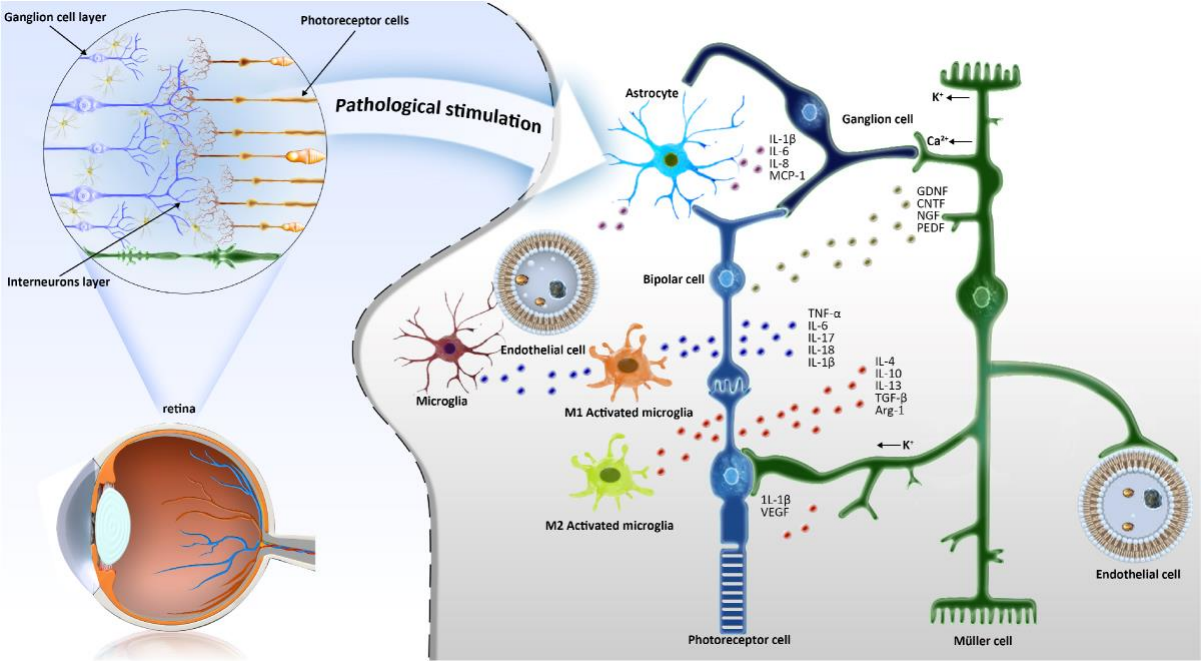
**Schematic diagram of the crosstalk between microglia and synaptic cells**. In normal retinal tissue, ramified microglia are mainly distributed in the ganglion cell layer, inner plexus layer and outer plexus layer. When stimulated by pathology, microglia cells react quickly and change into an activated state. The interaction between microglia and Müller cells forms a trophic factor control system in the process of retinal degeneration. GDNF, glial cell line-derived neurotrophic factor; CTNF, ciliary neurotrophic factor; NGF, nerve growth factor; PEDF, pigment epithelium-derived factors; VEGF, vascular endothelial growth factor; TGF-β, transforming growth factor-β; MCP-1, Monocyte chemoattractant protein-1.

Glial cells in the CNS will react in response to practically any kind of pathologic stimulus. Microglia, undergo a quite substantial change in their functional and structural phenotype when neurons in their immediate vicinity are damaged [101]. Müller cells demonstrate gliotic changes in response to pathological insults. microglia-Müller cell interactions appear to be a mode of bi-directional communications that help shape the overall injury response in the retina [90] (Fig. 3). Under normal circumstances, Müller cells constitute a potential source of extracellular ATP, mediating motor activity-dependent regulation of microglia dynamic processes. Müller cells secrete TSPO ligand that is expressed in microglia, and regulates microglia phagocytosis by inhibiting overactivation of microglia [102].

In order to ensure optimal homeostasis of retina, it is necessary to maintain a constant level of neurotrophic factors in retinal tissue. It has been shown that the interaction between microglia and Müller cells can act as a trophic factor control system in the process of retinal degeneration and may contribute to the protection of photoreceptors. When the microglia are over-activated by stress stimulus, they will send signals to Müller cells, and ask them to make some adjustments and promote their recovery in the event of injury [103]. However, prolonged interactions between microglial and Müller glial cell will become detrimental to retina and accelerate neuronal death. These chronic reactive Müller cells induce proliferative gliosis in degenerative retinas, and promote neuronal cell death by synthesizing and secreting TNF-α [102]. In addition to releasing chemotactic kinases, reactive Müller cells have been reported to provide adhesive cellular scaffolds that direct the movement of resident microglia across various retinal layers in the damaged retina [51]. A recent study also show that the bidirectional crosstalk promoted the further activation, migration and cell adhesion of microglia [104]. Researchers injected lipopolysaccharide into the vitreous cavity to increase the expressions of Müller-microglia cell adhesion molecules. This induction can lead to the radial displacement of activated microglia across retinal layers [51]. These adjustments would amplify the inflammatory response in the degenerative retina. Müller cell activation releases ATP via connexin 43 (CX43) semi-channels and activates microglia via ATP/P2X7 receptor (P2X7R) pathways [105, 106]. these Activated microglia in turn enhance the retinal inflammatory response induced by Müller cell activation, thus exacerbating retinal ganglion cell (RGC) loss in degenerative retina [107]. Collectively, these evidences suggest that the interaction between microglia and Müller cells can contribute to the retinal degeneration and the apoptosis of retinal neurons. P2X7R is a trimeric ATP-gated cation channel that can be activated by extracellular adenosine triphosphate (ATP). The activation of P2X7R leads to the release of pro-inflammatory mediators and cellular damage [108]. In glaucoma patients, extracellular ATP levels increase as intraocular pressure increases, adversely affecting the survival of RGC in retina [109]. Studies have shown that injecting benzoylbenzoyl-ATP (BzATP), a P2X7R agonist, into the vitreous cavity of mice can reduce the branch length of microglia and increases the cell body of microglia. the P2X7R activation occurs rapidly with the increase of intraocular pressure after stimulation [110]. In another in vitro study the P2X7R antagonist eye drops (JNJ47965567) were chronically applied on the DBA2J mouse, a genetic model of spontaneous glaucoma. The results showed that the P2X7R inhibition leads to the reduced number of activated microglia, enhanced survival of RGC, and the improved retinal electroretinogram (ERG)function [111]. SOD1 is a highly reactive oxidant which participates in the conversion of superoxide radicals into hydrogen peroxide and molecular oxygen, thereby shielding the cells from oxidative stress. Chronic oxidative stress and AMD-like features such as drusen, choroidal neovascularization, RPE dysfunction has been detected in mice with *Sod* knockout (*Sod1* KO mice). Carver et al. investigated whether concurrent knockout of P2X7R could block AMD-like pathological features in *Sod1* KO mice. The experimental results showed that P2X7R knockout saved the retinal thickness of *Sod1* KO mice, alleviated oxidative stress, and significantly reduced the number of activated microglia [68]. Furthermore, increased retinal microvascular permeability is recognized as a pathological hallmark in diabetic retinopathy (DR). researchers found that P2X7R mRNA and protein levels increased significantly in a Streptozotocin (STZ) -induced DR rat model, accompanied by the release of inflammatory cytokine IL-6. Intraperitoneal injection of P2X7R antagonist (AZ10606120 or A740063) reversed the defects in retinal vascular permeability and inhibit the release of inflammatory cytokines [112]. In this context, P2X7R plays an important role as a new pharmacological target in retinal diseases, which may have relevant clinical implications.

## Current therapeutics strategies targeting the neuroglia interaction

Current clinical trials have investigated drugs, biologics, and small molecules that target AMD pathogenesis, such as oxidative stress, visual cycle inhibition, complement activation, retinal/choroid blood flow, beta-amyloid protein (Aβ), and lipid accumulation (Table. 1). However, none of them that can halt completely the progression of AMD [113]. The microglia-Müller cell interaction appears to be a potential therapeutic target. In patients with AMD, microglia migrate to the photoreceptor layer and the inferior lumen of the retina, showing a strong proliferation enhancement. Activated microglia are found to kill photoreceptors in the outer retina in patients with advanced AMD [114]. Gliotic Müller cells display a dysregulation of various neuron-supportive functions. They induce disturbances of retinal glutamate metabolism and ion homeostasis, and cause the development of retinal edema and neuron death [115]. Therefore, regulating the activation phenotype of microglia and Müller cells may act as an effective control method for the treatment of AMD.

**Table 1:** Summary of therapeutic Compound/ Drugs for AMD.

Category	Compound/Drug	Targets	Experimental object	Reference
**Anti-inflammatory**	Methotrexate	the transmethylation products spermine, spermidine	Human	[117]
	Rapamycin	COX2	Human	[118]
	Infliximab	TNF-α	Human	[192]
	Tetracyclines	inhibit caspase activation	Human	[181]
	Minocycline	CCL2,IL-6, iNOS	BV-2 microglia cell	[121]
	Cortisone fluoride (FA), Triamcinolone acetonide (TA)	CCL2, IL-6, IL-8	Mouse, 661w cells, ARPE-19 cells	[122, 193]
	Epigallocatechin-3-gallate	HIF-1α/VEGF/VEGFR2	mouse	[127]
	Theissenolactone B (LB53)	NF-κB, ROS	ARPE-19 cells, mouse BV-2 cells, mouse	[60]
	Resveratrol (3,4',5 trihydroxy-trans-stilbene)	IL-6, IL-8	RPE cells	[129]
	Curcumin	reduces free radicals	ARPE-19 cell	[132]
**Complement system**	POT-4	C3	Human	[141]
	APL-2	C3	Human	[143]
	Efdamrofusp alfa	C3b, C4b	Human	[145]
	recombinant FH (recFH)	C3	mouse	[148]
**Antiapoptotic**	Centella asiatica extract (CA-HE50)	Nrf2/HO-1	ARPE-19 cells, mouse	[149]
	Delphinitin	Bax, caspase-3	ARPE-19 cells	[151]
	Shihu Yeguang Pill (SYP)	c-fos, c-jun, Bcl-2, TNF-α	Human, mouse	[153]
	Cordyceps militaris (CMCT)	NADPH oxidase-1 (NOX1), ROS	ARPE-19 cells	[155]
	Alpha-mangostin (α-mangostin, α-MG)	Bax, caspase-3, PI3K/AKT-PGC-1α/STRT-3	ARPE-19 cells	[158]
**Anti-VEGF**	Ranibizumab, Bevacizumab	All VEGF-A subtypes	Human	[169,173,194]
	Faricimab	VEGF-A, Ang-2	Human	[184,185]
	Pegaptanib	VEGF165	Human	[118]
	Aflibercept	VEGF-A, VEGF-B, PLGF	Human	[178]
	Brolucizumab	VEGF-A	Human	[188]

### Anti-inflammatory drug

Sustained inflammatory response promotes the pathological processes of degenerative retinopathy [116]. Anti-inflammatory drugs such as corticosteroids, non-steroidal anti-inflammatory drugs (NSAIDs), immunosuppressants (such as methotrexate and rapamycin), and biologic agents (such as Infliximab, daclizumab, and complement inhibitors) can provide an alternative therapeutic to inhibit the progression of AMD [117]. Tetracycline is a broad-spectrum antibiotic with anti-inflammatory effects. It can reduce ROS, inhibit caspase activation, prevent complement activation, and inhibit MMP. Recent study show that tetracycline can inhibit inflammatory cytokine production through their effects on microglia and T cell activation [118]. It has also been reported that tetracycline can target low-grade inflammation caused by abnormal complement pathway activation, which is thought to underlie the pathogenesis of AMD. Tetracycline may slow the progression of geographic atrophy (GA) [119]. Minocycline is a semi-synthetic tetracycline analogue that highly penetrates the blood-brain barrier and produces anti-inflammatory effects [120]. Rashid, K. et al. studied the effect of minocycline on BV-2 microglia in vitro. The results showed that minocycline significantly decreased the transcription of CCL2, IL-6 and iNOS in microglia. The immunomodulatory properties of minocycline on photoinduced retinal degeneration in mice were also investigated. In vivo experiments showed that minocycline downregulated the expression of microglia activation marker TSPO and CD68, and significantly reduced the number of activated microglia [121]. Moreover, Tanja Racic et al. have reported the anti-inflammatory effects of cortisone fludrocortisone (FA) and triamcinolone acetonide (TA) on the Müller cell-mediated inflammatory responses. After stimulating Müller glial cells with IL-1β or TNF-α, FA and TA can significantly reduce the expression of downstream inflammatory cytokines such as CCL2, IL-6 and IL-8 [122]. Another study show that TA reduces the Müller cells activation in mice received argon laser photocoagulation, as evidenced by the decreased number of infiltrating inflammatory cells [123]. Cellular retinaldehyde-binding protein (CRALBP) is expressed in RPE and Müller cells and binds to 11-cis-retinol and 11-cis-retina, thereby maintaining the chromophore supply of rod and cone photoreceptors. CRALBP is encoded by *Retinaldehyde-binding protein 1* (*RLBP1*) gene and mutations in *RLBP1* lead to an autosomal recessive form of retinal degeneration [124]. An animal study has shown that intravitreal injection of TA can reduce the phosphorylation level of p38 stress-activated protein kinase (p38/SAPK), inhibit the expression of c-Jun N-terminal kinase/stress-activated protein kinase (JNK/SAPK), thereby suppressing the neuroglia activation in *RLBP1-CreER-DTA176* transgenic mice [125, 126]. Collectively, these findings suggest that glucocorticoids may have potential value in the treatment of AMD. Some nature compounds also have the potential to be developed as an anti-AMD drug. For instance, pigallocatechin-3-gallate, can also alleviate laser-induced CNV leakage and reduces CNV area in AMD mice by down-regulating HIF-1α/VEGF/VEGFR2 pathway [127]. Lin FL et al. explored the theissenolactone B (LB53) induced effects on the sodium iodate (NaIO_3_)-induced retinal degeneration. They found that LB53 could protect retinal neuron function, improve retinal blood flow, and effectively reduce the photoreceptor loss in the NaIO_3_ induced AMD mouse model [60]. matrix metalloproteinase-9 (MMP-9) is expressed in Bruch’s membranes and RPEs, blood vessels (endothelial cells), and stroma. MMP-9 can enhance the release of inflammatory cytokines and chemokines [128]. In vitro studies showed that LB53 showed strong anti-MMP-9 activity in RPE cells and exerted strong anti-inflammatory effects on microglia [60]. Resveratrol (3,4',5 trihydroxy-trans-stilbene) is a plant phenolic with potent anti-inflammatory properties. Losso et al. find that resveratrol inhibits the production of inflammatory cytokines, such as IL-6 and IL-8, in the RPE cells following a dose-dependent manner [129]. Another in vitro study also shows that Carbon Monoxide-Releasing Molecules (CORMs) can activate the expression of genes associated with nuclear factor erythroid 2 associated factor 2 (Nrf2) in intense light- damaged RPE cells. CORMs significantly inhibit the expression of TNF-α induced intercellular adhesion molecule 1 and abolishes the VEGF-induced migration of endothelial cells [130]. Curcumin is a polyphenol compound extracted from turmeric root, which has antioxidant, anti-inflammatory, anti-mutagenesis, antibacterial and anticancer activities [131]. An in vitro study shows that curcumin reduces the expression of free radicals and enhances the expressions of SOD and glutathione (GSH) in a hydrogen peroxide-induced ARPE-19 cell senescence model [132]. Therefore, targeting neuroinflammation through anti-inflammatory strategies may slow the progression of AMD.

### Targeting the complement system

Complement system is a highly interacting network of immune proteins which are activated in cascades to neutralize microbial invaders or endogenous stress signals through potent opsonophagocytic and inflammatory mechanisms [133]. The mRNA of complement components, such as C1qb, C1r, C2, C3, C4, complement factor B (CFB) and CFH, are detected in the retina, RPE layer and choroid, indicating the complement regulatory system exists in these tissue [134]. Notably, genome-wide correlation studies have clearly shown that genetic variations in the complement system are associated with AMD pathogenesis. Retinal microglia and RPE cells are the main cellular sources of local complement expression. CD46, CD59 and CFH are detected in chorionic membranes of RPE cells [135]. Other studies show that complement 5a receptor (C5aR), a receptor of the complement system C5a, plays an important role in the inflammatory response and tissue regeneration [136]. C5aR is expressed in RPE cells, microglia, and Müller cells [134]. The relationship between complement and microglia is closed, because microglia act as resident immune sensors in the retina and can be rapidly transformed into reactive phagocytes following stimulus insults [11]. In patients with atrophic AMD, the deposition of complement (including C3) in the lesion promotes the disease progression [26]. C3-expressing microglia/macrophages and complement activation is found in the expanded area of external retinopathy in degenerative retinas. Thus far, the complement-mediated retinal inflammation has been well explored, with some preclinical and clinical studies suggesting that inhibiting specific complement pathways exerts a protective effect on degenerative retinopathy. For instance, Induction of CNV by laser damage is a classic method to establish Wet AMD animal model. Inhibition of C3a, C5a, CFB, and membrane attack complex (MAC) or the complement regulatory molecules CD59 and CFH can inhibit the occurrence of CNV in the laser induced AMD model [137-139]. Natoli R et al. demonstrated that vitreous delivery of small interfering RNA (siRNA) inhibits local expression of C3, thereby suppressing retinal complement activation and reducing the scale of retinal degeneration [140]. POT-4 compost protein is a cyclic peptide that inhibits C3 cracking and prevents complement activation.

A preliminary evaluation in a Phase I study of vitreous injection of POT-4 in AMD patients showed favorable effectiveness and safety [141]. The second-generation compstatin derivative Pegcetacoplan (APL-2) with a longer half-life. APL-2 is a cyclic peptide conjugated with polyethylene glycol polymer, which acts as an inhibitor for the C3 and prevents overactivation of complement system [142].

In the Phase II clinical trial (FIL-LY), GA lesion growth was 29% lower in the APL-2-treated group after intravitreal injection than in the control group. In a post hoc evaluation six months after the trial, APL-2-treated group showed a significant 47 percent reduction in lesion growth [143]. Macular-FAG, consisting primarily of a mixture of fatty acids, can ameliorate retinal degenerative events associated with advanced AMD by inhibiting the activation of C3 and C5 in the complement system, thereby reducing macrophage recruitment. Fatty acid based dietary supplement of Macular-FAG reduces retinal and RPE/choroid inflammation. Immunohistochemical results showed that Macular-FAG alleviated the retinal degeneration in a polyethylene glycol (PEG)-400 induced AMD model [144]. Efdamrofusp alfa is a bispecific fusion protein that binds to the VEGF isoform and complement factor C3b/C4b. A recent study has verified the safety, tolerability, and efficacy of efdamrofusp alfa in the treatment of wet AMD. 20 weeks after intravitreal injection of efdamrofusp alfa, the central subfield thickness (CST) and CNV area of treated eye decreased significantly [145]. CFH consists of 20 complement control protein (CCP) units, in which CCPs1-4 is responsible for the cofactor and accelerated degradation activity of CFH, while CCPs6-8 and CCPs19-20 carry two binding sites of glycosaminoglycan (gag) on the host cell and Bruch membrane [146]. CFH acts in body fluids and cell surfaces by preventing the formation of C3/C5 invertase and assisting the degradation of C3b through FI. Complement inhibition is to a large extent achieved by the FI induced proteolytic degradation of complement factors C3b and C4b [147]. CFH is an inhibitor that replaces the complement pathway and prevents overactivation of complement. Impairment of the regulatory function of CFH leads to the occurrence of inflammation, which is associated with the development and progression of AMD. In a mouse model nAMD, intravitreal injection of recombinant FH (recFH) reduced CNV area as effectively as anti-VEGF antibodies. recFH also reduces the deposition of microglia recruitment markers in C3 cut fragments, MAC, and CNV lesions [148]. These findings suggest that modulating the complement cascade activation provides potential reference value for the clinical treatment of AMD.

### Antiapoptotic drug

Caspase-dependent and caspase-independent pathways can lead to the loss of photoreceptors in the degenerative retina. Thus, anti-apoptotic drugs may provide another avenue for AMD treatment. Centella asiatica extract (CA-HE50) inhibits apoptosis cascades through Nrf2/HO-1 signaling pathway and increases the survival of retinal ARPE-19 cell line. In AMD mouse model, CA-HE50 significantly increases the thickness of ONL and preserves most of the ONL nuclei [149]. Delphinidin is a type of antioxidant drug that exhibits anti-inflammatory, antiproliferative, and antitumor activities in cancer cell lines [150]. A study shows that delphinidin effectively protects human ARPE-19 cells from oxidative damage induced by H_2_O_2_ through anti-apoptosis and antioxidant effects [151]. *Maternally expressed gene 3* (*MEG3*) silencing has a protective effect on photo-induced retinal degeneration and photoreceptor cell apoptosis in vivo. Mechanistically, *MEG3* regulates the function of retinal photoreceptor cells by acting as a p53 decoy. *MEG3* silencing reduces caspase 3/7 activity, up-regulates the expression of Bcl-2 and down-regulates the expression of Bax [152]. Shi hu Ye guang Pill (SYP) is a classic Chinese medicine formula with a long history of clinical application, which is widely used in the treatment of retinopathy in China. Hanhan Wu et al. showed that SYP treatment could reduce the structural and functional damage of retina caused by phototoxicity. Real-time PCR analysis showed that the abnormal expressions of pro-apoptotic c-fos and c-jun, anti-apoptotic Bcl-2 and pro-inflammatory TNF-α in damaged retina after SYP treatment [153]. Carotenoids exhibit natural immunomodulatory, antioxidative, antitumor, antiaging, and antibacterial properties, as evidenced by several related studies [154]. The carotenoids obtained from isolation and purification of Cordyceps militaris (CMCT) inhibited the production of H_2_O_2_-induced ROS and the protein expression of NADPH oxidase-1 (NOX1). CMCT improved the levels of malondialdehyde (MDA), decreased the expressions of catalase (CAT), SOD and GSH in cells induced by oxidative stress. CMCT protects ARPE-19 cells from oxidative stress-induced damage through its antioxidant activity and anti-apoptotic function, suggesting the potential therapeutic role of CMCT in the prevention and remission of AMD [155]. miR-155-5p targets the following cell cycle and proliferation-related genes: cyclin-dependent kinases2 (CDK2), cyclin-dependent kinases4(CDK4), cyclin D1(CCND1), and cyclin D2(CCND2). mini-αA is a functional fragment of αA-crystallin with molecular chaperone activity [156]. A recent study showed that the expression of miR-155-5p was up-regulated in NaIO_3_-induced cells, while down-regulated in mini-αA treated cells. Dual luciferase reporter assay was used to verify the targeting binding relationship between miR-155-5p and CDK2. The results showed that mini-αA could reverse the oxidative damage and apoptosis induced by NaIO_3_. miR-155-5p is involved in the anti-oxidative damage and anti-apoptotic effects of mini-αA through CDK2 regulation [157]. Chuang, C.J. et al. demonstrated that alpha-mangostin (α-mangostin, α-MG) has inhibitory effects on NaIO_3_-induced ROS dependent toxicity in vivo and in vitro. α-MG significantly increased cell viability and reduced cell apoptosis induced by NaIO_3_-induced oxidative stress by inhibiting Bax, Poly (ADP-ribose) (PAR) polymerase-1 (PARP-1), cleaved caspase-3 and enhancing Bcl-2 protein expression. In vivo studies showed that α-MG improved retinal morphological structure, increased the thickness of the outer and inner layers by suppressing cleaved caspase-3 expression [158]. The study of anti-apoptosis mechanism may provide more strategies for clinical treatment of retinal diseases.

### Stem Cell Therapy

Stem cells are infinitely self-renewing cells that can differentiate into other cell types. Induced pluripotent stem cells (iPSCs) and hESCs can differentiate into retinal cells and have the same characteristics as the original cells at both genetic and functional levels. The first clinical trials of hESCs and iPSCs from patients with AMD were conducted in the US and Japan. Preliminary and phase I/II clinical studies showed that hESCs -derived RPE cell therapy is safe and effective in promoting central and peripheral vision in patients with AMD [159, 160]. Da Cruz, L. et al. developed a therapeutic, biocompatible hESC-RPE monolayer on a coated synthetic membrane, herein termed a “patch”. Then, an RPE patch is placed in the subretinal space using a surgical delivery tool. Two patients' vision improved by 29 and 21 letters over 12 months, respectively [161]. These new technologies help improve vision and provide novel treatment strategies for AMD. Subretinal transplantation of hESCs -derived RPE cells was performed in a preclinical mouse AMD model. Seven months after treatment some injected cells formed RPE monolayers above the primary layer [162]. In a similar study, retinal progenitor cells (RPCs) from hESCs were integrated into the GCL of mice, expressing the RGCs marker Brn3a, and the ONL of injected animals was accompanied by an increase in thickness [163]. In a retinitis pigmentosa (RP) mouse model, subretinal transplantation of iPSC-derived RPE spheroids delayed thinning of retinal ONL, increased PEDF levels, reduced the number of apoptotic cells as well as microglia infiltration in the retina [164]. In 2019, Banin et al. reported in an interim report on the Phase I/IIa trial (NCT 02286089) of 12 patients with advanced dry AMD and GA that when administered in combination with systemic immunosuppression prior to transplantation, hESC-derived RPE cells were well tolerated in patients. Transplanted cells were detected in the inferior lumen of the retina and improvements in the RPE layer along the GA margin were observed. In 2020, they updated Group 4 of the Phase I/IIa trial (NCT 02286089) to report improved vision and changes in the appearance of glass warts in treated patients. Preretinal membrane (ERM) formation and retinal detachment were observed in a few patients, all of whom were successfully treated [165]. Challenges in stem cell therapy in addition to the protective and regenerative effects of stem cell transplantation in retinal diseases, several clinical and preclinical studies have reported transplant-related adverse effects. For example, in a rat model of anterior ischemic optic neuropathy, vitreous transplantation of human Wharton’s jelly mesenchymal stem cell (WJ-MSCs) induced retinal damage and severe inflammation with macrophage infiltration. However, vitreous injection of conditioned medium (CM) from Mesenchymal Stem Cell (MSC) inhibited apoptosis of RGCs and reduced inflammation by inhibiting microglia activation and macrophage infiltration [166]. However, the disadvantage of stem cell therapy is that it takes a lot of time and money.

### Anti-vascular endothelial growth factor (VEGF) drugs

Anti-VEGF-A antagonists, such as ranibizumab, bevacizumab, pegaptanib and aflibercept, have been used for the treatments of wet AMD in clinical practice. Vitreous anti-VEGF drugs inhibit the functional activity of pro-angiogenesis factors with different targeting selectivity, affinity and potency [167-169]. Pegaptanib is a pegylated oligoribonucleotide that binds to VEGF165 with high specificity and affinity. It is the first intravitreal anti-VEGF therapy specifically developed for nAMD [170]. Two simultaneous, multi-centers, prospective, randomized, double-blind and controlled clinical trials were conducted on AMD patients who have received a 48-week pegaptanib treatment. During the treatment, three intravitreal doses of pegaptanib (0.3mg, 1.0mg, and 3.0mg) were given every 6 weeks. Results of the efficacy analysis showed that a significantly higher proportion of patients maintained or improved visual acuity in the Pegaptanib treatment group compared to the sham injection group [171].

Bevacizumab is the first approved angiogenesis inhibitor that targets VEGF-A monoclonal antibody fragments [172]. Bevacizumab can reduce the expression of genes related to angiogenesis, apoptosis, inflammation and oxidative stress in a variety of cell lines [173]. Bevacizumab, used as an off-label drug, has been shown to be effective in treating nAMD [174]. In a clinical study, Bevacizumab (0.5mg) was injected intravitreal into 18 eyes of nAMD patients, and visual ability was examined one month after injection and three months after injection. The results showed that changes in central foveal thickness (CFT) were improved after intravitreal injection of bevacizumab. Mean retinal sensitivity in the macular field improved significantly 1 month after anti-VEGF. The results indicate that bevacizumab is effective in treating nAMD [175].

Ranibizumab and bevacizumab are angiogenesis inhibitors that target VEGF-A monoclonal antibody fragments. In multiple clinical trials and long-term studies, ranibizumab has shown its good efficacy and safety for the treatment of CNV [176]. In the MARINA (Minimally Classic/Occult Trial of the Anti-VEGF Antibody Ranibizumab in the Treatment of Neovascular AMD) and ANCHOR (Anti-VEGF Antibody for the Treatment of Predominantly Classic Choroidal Neovascularization in AMD) trials, it was demonstrated that about 88% of patients treated with monthly intravitreal injections of ranibizumab had improved vision after two years [169]. Aflibercept is a fusion protein containing the second Ig domain of human VEGF receptor (VEGFR). It can fuse with the constant region (Fc) of human IgG1 (115 kDa) [177]. Aflibercept captures VEGF-A, VEGF-B and placental growth factor (PlGF), thereby improving the visual acuity and macular morphology in a large number of treatment-naive eyes with polypoidal choroidal vasculopathy (PCV) [178]. PlGF is a multifunctional peptide that contributes to the angiogenesis pathology in several ocular diseases. For instance, PlGF level in the vitreous body of DR patient is positively correlated with degree of retinal ischemia [179]. Lazzara et al. have demonstrated that aflibercept and anti-PLGF antibodies protect human retinal endothelial cells (HRECs) and mouse RPEs from high glucose damage by blocking the ERK pathway activation and the subsequent TNF-α release. Another experiment showed that the level of TNF-α protein in the retinal tissue of diabetic rat reduced significantly after intravitreal injection of aflibercept [180]. Aflibercept also can modulate the retinal inflammation in oxygen-induced retinopathy (OIR) mouse models. Retinas of OIR mice treated with aflibercept showed an increased number of branching microglia (the "resting" state) and a reduced number of the activated microglia compared with untreated controls. Taken together, these findings suggest that aflibercept acts an effective modulator of retinal inflammation in degenerative retinopathy [181].

Faricimab (faricimab-svoa; Vabysmo™) is a bi-specific antibody that binds to a VEGF-A and angiopoietin-2 (Ang-2). Faricimab was first approved in the United States for the treatment of retinal neovascular disease in 2022 [182]. It is designed to utilize the specific heterodimerization of two different antigen-binding domains. Its Fc domain has been optimized to eliminate binding interactions with neonatal Fc and Fc gamma receptors, which reduce the antibody's systemic half-life and the likelihood of inflammatory side effects [183]. In two randomized, double-blind, Phase III, non-adverse trials, patients over 50 years of age who were initially treated with nAMD were randomly assigned (1:1) to receive intravitreal injection of faricimab 6.0 mg up to every 16 weeks or intravitreal injection of aflibercept 2.0 mg every 8 weeks. Nine months later, in TENAYA results, patients treated with faricimab had a mean change in best-corrected visual acuity (BCVA) of 5.8 letters from baseline correction; Patients treated with aflibercept corrected for an average change of 5.1 letters. In the LUCERNE trial, patients treated with faricimab and aflibercept both experienced an average change in BCVA of 6.6 letters from baseline correction after eight months. The mean change in BCVA from baseline for faricimab administered at a fixed interval of up to every 16 weeks was no worse than for aflibercept every 8 weeks. These findings suggest that faricimab prolonged the treatment interval in nAMD patients by dual inhibition of VEGF-A and Ang-2 [184]. Another BOULEVARD Phase II trial (NCT02699450) compared the safety and efficacy of faricimab with ranibizumab in patients with diabetic macular edema (DME). Enrolled patients were randomly assigned in a 1:1:1 ratio to intravitreal injections of 6.0 mg faricimab, 1.5 mg faricimab, and 0.3 mg ranibizumab. Patients were dosed once a month for 20 weeks, followed by an observation period of up to 36 weeks to assess durability. Results of the trial showed that 6.0 mg faricimab, 1.5 mg faricimab, and 0.3 mg ranibizumab resulted in an average improvement of 13.9, 11.7, and 10.3 Early Treatment Diabetic Retinopathy Study (ETDRS) letters from baseline in patients treated initially. The 6.0-mg faricimab dose showed a statistically significant gain of 3.6 letters relative to 0.3mg ranibizumab. Most importantly, in initial treatment patients, faricimab showed a statistically significant visual gain compared to ranibizumab in the 24th week. These faricimab treated patient had reduced CST, improved Diabetic Retinopathy Severity Scale (DRSS) scores, and prolonged lasting outcomes [185].

Brolucizumab, recently approved in the United States for the treatment of wet AMD, is the first single chain antibody fragment (scFv) specifically designed for intraocular use in human patients [186]. Brolucizumab inhibits all subtypes of VEGF-A, which helps prevent the growth and leakage of these abnormal blood vessels, improving vision in patients. Due to its small size, brolucizumab exhibits good tissue permeability within the eye and is able to reach high concentrations in the retinal tissue [187]. The development of brolucizumab overcomes the technical challenges associated with the design of a scFv for therapeutic applications and marks an important milestone in the focus on science. A Phase I/II SEE study (NCT01304693) evaluated the safety and efficacy of brolucizumab (single intravitreal injection of 4.0/6.0mg) versus ranibizumab (single intravitreal injection of 0.5mg) in 194 treated patients with nAMD. The results of the trial showed that brolucizumab was not inferior to ranibizumab in terms of mean CST changes. Moreover, brolucizumab had a longer duration of action than ranibizumab [188]. Anti-VEGF drugs may reduce systemic side effects by providing small doses in the vitreous cavity, however, they also can penetrate the blood circulation and alter VEGF throughout the body with unknown clinical consequences. In particular, the pharmacokinetics of anti-VEGF drugs in vitreous show significant interspecific differences. A clinical trial evaluated the systemic pharmacokinetics of aflibercept, bevacizumab, and ranibizumab in patients with degenerative retinopathy. Patients received intravitreal injections of aflibercept 2.0 mg, bevacizumab 1.25 mg, or ranibizumab (AMD/RVO 0.5 mg, DME 0.3 mg), respectively. The main outcome measures were serum PKs and plasma free-VEGF concentrations after the injections. It is shown that systemic exposure to each drug was highest with bevacizumab, then aflibercept, and lowest with ranibizumab. Ranibizumab cleared from the bloodstream more quickly than bevacizumab or aflibercept. Aflibercept treatment resulted in the greatest reductions in plasma free-VEGF relative to baseline levels, whereas ranibizumab treatment resulted in the smallest decreases in plasma free-VEGF [189]. Therefore, the clinical pharmacokinetics of ocular anti-VEGF agents should be well programmed when selecting appropriate agents for individual patients. Although anti-VEGF drugs can induce significant regression of CNV and preserve central vision, but their half-life is short and multiple intravitreal injections are required to achieve sustained therapeutic effects [190]. Repeated intravitreal injections increase the risk of multiple complications and adverse effects, such as endophthalmitis, retinal detachment, and iatrogenic traumatic cataract [191]. Moreover, VEGF-A antagonists do not work for all patients with wet AMD. The long-term safety of chronic anti-VEGF therapy is unclear, and chronic anti-VEGF therapy may impair the function of normal retinal vessels and cells.

## Outlook

AMD is the leading cause of irreversible blindness in aged population. The consequences of poor vision include an increased risk of falls, depression, and the need for long-term care when patients are unable to perform activities during daily living. Although advances have been made in the treatment of neovascular AMD, there is no effective treatment for the more common dry AMD. The crosstalk between microglia and Müller cells accelerates the onset and progression of AMD, but the exact pathogenesis is not clear. Elucidating the triggers and mechanism underlying the multicellular interactions is key to developing therapeutic target for AMD. Some novel molecular mediators targeting microglia to inhibit neurodegeneration are shown to slow the development of AMD by reducing inflammation and oxidative stress. However, they are not yet ready for application in clinical treatment and means of monitoring efficacy are not yet abundant. The next step requires more thorough understandings of the retinal inflammatory response, retinal glial activation, the link between microglia and Müller cells, the temporal development sequence of glia and neuronal alterations. Therefore, future studies on the molecular mechanisms involved in the interaction between microglia and Müller cells may provide additional aids for development novel therapy for AMD.

## References

[1] Zhang Y, Erhard AL, Plagemann T, Eter N, Heiduschka P (2021). A modified protocol for isolation of retinal microglia from the pig. Exp Eye Res, 207:108584. 33910034 10.1016/j.exer.2021.108584

[2] Li J, Yu S, Lu X, Cui K, Tang X, Xu Y, et al. (2021). The phase changes of M1/M2 phenotype of microglia/macrophage following oxygen-induced retinopathy in mice. Inflammation Research, 70:183-192. 33386422 10.1007/s00011-020-01427-w

[3] Monk PN SP (2006). ALS: life and death in a bad neighborhood. Nat Med, 12:885-887. 16892030 10.1038/nm0806-885

[4] Butovsky O, Talpalar AE, Ben-Yaakov K, Schwartz M (2005). Activation of microglia by aggregated beta-amyloid or lipopolysaccharide impairs MHC-II expression and renders them cytotoxic whereas IFN-gamma and IL-4 render them protective. Mol Cell Neurosci, 29:381-393. 15890528 10.1016/j.mcn.2005.03.005

[5] Zhou X, Spittau B, Krieglstein K (2012). TGFβ signalling plays an important role in IL4-induced alternative activation of microglia. J Neuroinflammation, 9:210. 22947253 10.1186/1742-2094-9-210 PMC3488564

[6] Bringmann A, Wiedemann P (2009). Involvement of Müller glial cells in epiretinal membrane formation. Graefes Arch Clin Exp Ophthalmol, 247:865-883. 19415318 10.1007/s00417-009-1082-x

[7] Wong WL, Su X, Li X, Cheung CM, Klein R, Cheng CY, et al. (2014). Global prevalence of age-related macular degeneration and disease burden projection for 2020 and 2040: a systematic review and meta-analysis. Lancet Glob Health, 2:e106-116. 25104651 10.1016/S2214-109X(13)70145-1

[8] Hamid MA, Moustafa MT, Nashine S, Costa RD, Schneider K, Atilano SR, et al. (2021). Anti-VEGF Drugs Influence Epigenetic Regulation and AMD-Specific Molecular Markers in ARPE-19 Cells. Cells, 10:878. 33921543 10.3390/cells10040878 PMC8069662

[9] Zhao T, Guo X, Sun Y (2021). Iron Accumulation and Lipid Peroxidation in the Aging Retina: Implication of Ferroptosis in Age-Related Macular Degeneration. Aging Dis, 12:529-551. 33815881 10.14336/AD.2020.0912 PMC7990372

[10] Heiduschka P, Eter N, Uhlig CE, Grune T, Höhn A, König J, et al. (2023). Sub-Retinal Injection of Human Lipofuscin in the Mouse - A Model of “Dry” Age-Related Macular Degeneration? Aging and disease, 14:184-203. 36818570 10.14336/AD.2022.0626 PMC9937713

[11] Wang M, Wong WT (2014). Microglia-Muller cell interactions in the retina. Adv Exp Med Biol, 801:333-338. 24664715 10.1007/978-1-4614-3209-8_42 PMC4685688

[12] Blasiak J (2020). Senescence in the pathogenesis of age-related macular degeneration. Cell Mol Life Sci, 77:789-805. 31897543 10.1007/s00018-019-03420-x PMC11105088

[13] Rudnicka AR JZ, Wormald R, Cook DG, Fletcher A, Owen CG. (2012). Age and gender variations in age-related macular degeneration prevalence in populations of European ancestry: a meta-analysis. Ophthalmology, 119:571-580. 22176800 10.1016/j.ophtha.2011.09.027

[14] Malek G LC, Guidry C, Medeiros NE, Curcio CA. (2003). Apolipoprotein B in cholesterol-containing drusen and basal deposits of human eyes with age-related maculopathy. Am J Pathol, 162:413-425. 12547700 10.1016/S0002-9440(10)63836-9 PMC1851166

[15] Handa JT, Bowes Rickman C, Dick AD, Gorin MB, Miller JW, Toth CA, et al. (2019). A systems biology approach towards understanding and treating non-neovascular age-related macular degeneration. Nat Commun, 10:3347. 31350409 10.1038/s41467-019-11262-1 PMC6659646

[16] Chatziralli I, Theodossiadis G, Panagiotidis D, Pousoulidi P, Theodossiadis P (2018). Choriocapillaris Vascular Density Changes in Patients with Drusen: Cross-Sectional Study Based on Optical Coherence Tomography Angiography Findings. Ophthalmol Ther, 7:101-107. 29383674 10.1007/s40123-018-0119-9 PMC5997591

[17] Zhao H, Roychoudhury J, Doggett TA, Apte RS, Ferguson TA (2013). Age-Dependent Changes in FasL (CD95L) Modulate Macrophage Function in a Model of Age-Related Macular Degeneration. Investigative Opthalmology & Visual Science, 54:5321-5331. 10.1167/iovs.13-12122 PMC3738220 23821188

[18] Elzey BD GT, Herndon JM, Barreiro R, Tschopp J, Ferguson TA. (2001). Regulation of Fas ligand-induced apoptosis by TNF. J Immunol, 167:3049-3056. 11544288 10.4049/jimmunol.167.6.3049

[19] Chen M, Xu H (2015). Parainflammation, chronic inflammation, and age-related macular degeneration. J Leukoc Biol, 98:713-725. 26292978 10.1189/jlb.3RI0615-239R PMC4733662

[20] Ebrahimi KB, Handa JT (2011). Lipids, lipoproteins, and age-related macular degeneration. J Lipids, 2011:802059. 21822496 10.1155/2011/802059 PMC3147126

[21] Hageman GS, Luthert PJ, Victor Chong NH, Johnson LV, Anderson DH, Mullins RF (2001). An integrated hypothesis that considers drusen as biomarkers of immune-mediated processes at the RPE-Bruch's membrane interface in aging and age-related macular degeneration. Prog Retin Eye Res, 20:705-732. 11587915 10.1016/s1350-9462(01)00010-6

[22] Telander DG (2011). Inflammation and age-related macular degeneration (AMD). Semin Ophthalmol, 26:192-197. 21609232 10.3109/08820538.2011.570849

[23] Medzhitov R (2008). Origin and physiological roles of inflammation. Nature, 454:428-435. 18650913 10.1038/nature07201

[24] Szatmari-Toth M, Ilmarinen T, Mikhailova A, Skottman H, Kauppinen A, Kaarniranta K, et al. (2019). Human Embryonic Stem Cell-Derived Retinal Pigment Epithelium-Role in Dead Cell Clearance and Inflammation. Int J Mol Sci, 20:926. 30791639 10.3390/ijms20040926 PMC6412543

[25] Cipriani V, Leung HT, Plagnol V, Bunce C, Khan JC, Shahid H, et al. (2012). Genome-wide association study of age-related macular degeneration identifies associated variants in the TNXB-FKBPL-NOTCH4 region of chromosome 6p21.3. Hum Mol Genet, 21:4138-4150. 22694956 10.1093/hmg/dds225 PMC3428154

[26] Fritsche LG, Igl W, Bailey JN, Grassmann F, Sengupta S, Bragg-Gresham JL, et al. (2016). A large genome-wide association study of age-related macular degeneration highlights contributions of rare and common variants. Nat Genet, 48:134-143. 26691988 10.1038/ng.3448 PMC4745342

[27] Seddon JM, Ajani UA, Mitchell BD (1997). Familial aggregation of age-related maculopathy. Am J Ophthalmol, 123:199-206. 9186125 10.1016/s0002-9394(14)71036-0

[28] Klaver CC, Wolfs RC, Assink JJ, van Duijn CM, Hofman A, de Jong PT (1998). Genetic risk of age-related maculopathy. Population-based familial aggregation study. Arch Ophthalmol, 116:1646-1651. 9869796 10.1001/archopht.116.12.1646

[29] Fisher SA, Abecasis GR, Yashar BM, Zareparsi S, Swaroop A, Iyengar SK, et al. (2005). Meta-analysis of genome scans of age-related macular degeneration. Hum Mol Genet, 14:2257-2264. 15987700 10.1093/hmg/ddi230

[30] Klein RJ, Zeiss C, Chew EY, Tsai JY, Sackler RS, Haynes C, et al. (2005). Complement factor H polymorphism in age-related macular degeneration. Science, 308:385-389. 15761122 10.1126/science.1109557 PMC1512523

[31] Hallam TM, Marchbank KJ, Harris CL, Osmond C, Shuttleworth VG, Griffiths H, et al. (2020). Rare Genetic Variants in Complement Factor I Lead to Low FI Plasma Levels Resulting in Increased Risk of Age-Related Macular Degeneration. Invest Ophthalmol Vis Sci, 61:18. 10.1167/iovs.61.6.18 PMC7415286 32516404

[32] Lu F, Liu S, Hao Q, Liu L, Zhang J, Chen X, et al. (2018). Association Between Complement Factor C2/C3/CFB/CFH Polymorphisms and Age-Related Macular Degeneration: A Meta-Analysis. Genet Test Mol Biomarkers, 22:526-540. 30179527 10.1089/gtmb.2018.0110

[33] Gliem M, Müller PL, Mangold E, Holz FG, Bolz HJ, Stöhr H, et al. (2015). Sorsby Fundus Dystrophy: Novel Mutations, Novel Phenotypic Characteristics, and Treatment Outcomes. Invest Ophthalmol Vis Sci, 56:2664-2676. 25766588 10.1167/iovs.14-15733

[34] Allikmets R, Shroyer NF, Singh N, Seddon JM, Lewis RA, Bernstein PS, et al. (1997). Mutation of the Stargardt disease gene (ABCR) in age-related macular degeneration. Science, 277:1805-1807. 9295268 10.1126/science.277.5333.1805

[35] Guo C, Sun L, Chen X, Zhang D (2013). Oxidative stress, mitochondrial damage and neurodegenerative diseases. Neural Regen Res, 8:2003-2014. 25206509 10.3969/j.issn.1673-5374.2013.21.009 PMC4145906

[36] Jabbehdari S, Handa JT (2021). Oxidative stress as a therapeutic target for the prevention and treatment of early age-related macular degeneration. Surv Ophthalmol, 66:423-440. 32961209 10.1016/j.survophthal.2020.09.002

[37] SanGiovanni JP AD, Iyengar SK, Elashoff M, Clemons TE, Reed GF, Henning AK, Sivakumaran TA, Xu X, DeWan A, Agrón E, Rochtchina E, Sue CM, Wang JJ, Mitchell P, Hoh J, Francis PJ, Klein ML, Chew EY, Chakravarti A. (2009). Mitochondrial DNA variants of respiratory complex I that uniquely characterize haplogroup T2 are associated with increased risk of age-related macular degeneration. PLoS One, 4:e5508. 19434233 10.1371/journal.pone.0005508 PMC2677106

[38] Ambati J, Fowler BJ (2012). Mechanisms of age-related macular degeneration. Neuron, 75:26-39. 22794258 10.1016/j.neuron.2012.06.018 PMC3404137

[39] Amaral J, Lee JW, Chou J, Campos MM, Rodríguez IR (2013). 7-Ketocholesterol induces inflammation and angiogenesis in vivo: a novel rat model. PLoS One, 8:e56099. 23409131 10.1371/journal.pone.0056099 PMC3568027

[40] Kodjikian L, Mehanna CJ, Cohen SY, Devin F, Razavi S, Querques G, et al. (2021). The role of future treatments in the management of neovascular age-related macular degeneration in Europe. Eur J Ophthalmol, 31:2179-2188. 34053331 10.1177/11206721211018348

[41] Datta S, Cano M, Ebrahimi K, Wang L, Handa JT (2017). The impact of oxidative stress and inflammation on RPE degeneration in non-neovascular AMD. Prog Retin Eye Res, 60:201-218. 28336424 10.1016/j.preteyeres.2017.03.002 PMC5600827

[42] Sobrin L, Seddon JM (2014). Nature and nurture- genes and environment- predict onset and progression of macular degeneration. Prog Retin Eye Res, 40:1-15. 24374240 10.1016/j.preteyeres.2013.12.004 PMC6446565

[43] Rajapakse D, Curtis T, Chen M, Xu H (2017). Zinc Protects Oxidative Stress-Induced RPE Death by Reducing Mitochondrial Damage and Preventing Lysosome Rupture. Oxid Med Cell Longev, 2017:6926485. 29348791 10.1155/2017/6926485 PMC5733978

[44] Rowan S, Jiang S, Korem T, Szymanski J, Chang ML, Szelog J, et al. (2017). Involvement of a gut-retina axis in protection against dietary glycemia-induced age-related macular degeneration. Proc Natl Acad Sci U S A, 114:E4472-e4481. 28507131 10.1073/pnas.1702302114 PMC5465926

[45] Chew EY, Clemons TE, Sangiovanni JP, Danis RP, Ferris FL 3rd, Elman MJ, et al. (2014). Secondary analyses of the effects of lutein/zeaxanthin on age-related macular degeneration progression: AREDS2 report No. 3. JAMA Ophthalmol, 132:142-149. 24310343 10.1001/jamaophthalmol.2013.7376 PMC4636082

[46] Seddon JM, Cote J, Rosner B (2003). Progression of age-related macular degeneration: association with dietary fat, transunsaturated fat, nuts, and fish intake. Arch Ophthalmol, 121:1728-1737. 14662593 10.1001/archopht.121.12.1728 PMC8443211

[47] Chen JL, Hung CT, Keller JJ, Lin HC, Wu YJ (2019). Proteomic analysis of retinal pigment epithelium cells after exposure to UVA radiation. BMC Ophthalmol, 19:168. 31375076 10.1186/s12886-019-1151-9 PMC6679551

[48] Hao X CJ, Zhang Z. (2018). Polymorphisms in PEDF linked with the susceptibility to age-related macular degeneration: A case-control study. Medicine (Baltimore), 97:e11981. 30142832 10.1097/MD.0000000000011981 PMC6113020

[49] Garaschuk O, Verkhratsky A (2019). Physiology of Microglia. Methods Mol Biol, 2034:27-40. 31392675 10.1007/978-1-4939-9658-2_3

[50] Yoshida S, Yoshida A, Ishibashi T (2004). Induction of IL-8, MCP-1, and bFGF by TNF-alpha in retinal glial cells: implications for retinal neovascularization during post-ischemic inflammation. Graefes Arch Clin Exp Ophthalmol, 242:409-413. 15029502 10.1007/s00417-004-0874-2

[51] Wang M, Ma W, Zhao L, Fariss RN, Wong WT (2011). Adaptive Müller cell responses to microglial activation mediate neuroprotection and coordinate inflammation in the retina. J Neuroinflammation, 8:173. 22152278 10.1186/1742-2094-8-173 PMC3251543

[52] Yu C, Roubeix C, Sennlaub F, Saban DR (2020). Microglia versus Monocytes: Distinct Roles in Degenerative Diseases of the Retina. Trends Neurosci, 43:433-449. 32459994 10.1016/j.tins.2020.03.012 PMC7556353

[53] Liang KJ LJ, Wang YD, Ma W, Fontainhas AM, Fariss RN, Wong WT. (2009). Regulation of dynamic behavior of retinal microglia by CX3CR1 signaling. Invest Ophthalmol Vis Sci, 50:4444-4451. 19443728 10.1167/iovs.08-3357 PMC2749316

[54] Tang Y, Le W (2016). Differential Roles of M1 and M2 Microglia in Neurodegenerative Diseases. Mol Neurobiol, 53:1181-1194. 25598354 10.1007/s12035-014-9070-5

[55] Colton C WD (2010). Assessing activation states in microglia. CNS Neurol Disord Drug Targets, 9:174-191. 20205642 10.2174/187152710791012053

[56] Li R, Huang YG, Fang D, Le WD (2004). (-)-Epigallocatechin gallate inhibits lipopolysaccharide-induced microglial activation and protects against inflammation-mediated dopaminergic neuronal injury. J Neurosci Res, 78:723-731. 15478178 10.1002/jnr.20315

[57] Ponomarev ED, Maresz K, Tan Y, Dittel BN (2007). CNS-derived interleukin-4 is essential for the regulation of autoimmune inflammation and induces a state of alternative activation in microglial cells. J Neurosci, 27:10714-10721. 17913905 10.1523/JNEUROSCI.1922-07.2007 PMC6672829

[58] Ma W, Wong WT (2016). Aging Changes in Retinal Microglia and their Relevance to Age-related Retinal Disease. Adv Exp Med Biol, 854:73-78. 26427396 10.1007/978-3-319-17121-0_11 PMC4696750

[59] Natoli R, Fernando N, Madigan M, Chu-Tan JA, Valter K, Provis J, et al. (2017). Microglia-derived IL-1beta promotes chemokine expression by Muller cells and RPE in focal retinal degeneration. Mol Neurodegener, 12:31. 28438165 10.1186/s13024-017-0175-y PMC5404662

[60] Lin FL, Cheng YW, Chen LH, Ho JD, Yen JL, Wang MH, et al. (2023). Retinal protection by fungal product theissenolactone B in a sodium iodate-induced AMD model through targeting retinal pigment epithelial matrix metalloproteinase-9 and microglia activity. Biomed Pharmacother, 158:114138. 36535199 10.1016/j.biopha.2022.114138

[61] Fu X, Feng S, Qin H, Yan L, Zheng C, Yao K (2023). Microglia: The breakthrough to treat neovascularization and repair blood-retinal barrier in retinopathy. Front Mol Neurosci, 16:1100254. 36756614 10.3389/fnmol.2023.1100254 PMC9899825

[62] Hikage F, Lennikov A, Mukwaya A, Lachota M, Ida Y, Utheim TP, et al. (2021). NF-kappaB activation in retinal microglia is involved in the inflammatory and neovascularization signaling in laser-induced choroidal neovascularization in mice. Exp Cell Res, 403:112581. 33811906 10.1016/j.yexcr.2021.112581 PMC8479856

[63] Palazzo I, Kelly L, Koenig L, Fischer AJ (2023). Patterns of NFkB activation resulting from damage, reactive microglia, cytokines, and growth factors in the mouse retina. Experimental Neurology, 359:114233. 36174748 10.1016/j.expneurol.2022.114233 PMC9722628

[64] Wu J, Gao G, Shi F, Xie H, Yang Q, Liu D, et al. (2021). Activated microglia-induced neuroinflammatory cytokines lead to photoreceptor apoptosis in Abeta-injected mice. J Mol Med (Berl), 99:713-728. 33575853 10.1007/s00109-021-02046-6

[65] Khan AS, Langmann T (2020). Indole-3-carbinol regulates microglia homeostasis and protects the retina from degeneration. J Neuroinflammation, 17:327. 33143743 10.1186/s12974-020-01999-8 PMC7640677

[66] Scholz R, Caramoy A, Bhuckory MB, Rashid K, Chen M, Xu H, et al. (2015). Targeting translocator protein (18 kDa) (TSPO) dampens pro-inflammatory microglia reactivity in the retina and protects from degeneration. Journal of Neuroinflammation, 12:201. 26527153 10.1186/s12974-015-0422-5 PMC4630900

[67] Garcia-Garcia J, Usategui-Martin R, Sanabria MR, Fernandez-Perez E, Telleria JJ, Coco-Martin RM (2022). Pathophysiology of Age-Related Macular Degeneration: Implications for Treatment. Ophthalmic Research, 65:615-636. 35613547 10.1159/000524942

[68] Carver KA, Lin CM, Bowes Rickman C, Yang D (2017). Lack of the P2X(7) receptor protects against AMD-like defects and microparticle accumulation in a chronic oxidative stress-induced mouse model of AMD. Biochem Biophys Res Commun, 482:81-86. 27810364 10.1016/j.bbrc.2016.10.140 PMC5195873

[69] Karlstetter M NC, Aslanidis A, Moeller K, Horn F, Scholz R, Neumann H, Weber BH, Rupprecht R, Langmann T. (2014). Translocator protein (18 kDa) (TSPO) is expressed in reactive retinal microglia and modulates microglial inflammation and phagocytosis. J Neuroinflammation, 8:3. 10.1186/1742-2094-11-3 PMC3895821 24397957

[70] Dixon MA, Greferath U, Fletcher EL, Jobling AI (2021). The Contribution of Microglia to the Development and Maturation of the Visual System. Front Cell Neurosci, 15:659843. 33967697 10.3389/fncel.2021.659843 PMC8102829

[71] Bermond K, Wobbe C, Tarau IS, Heintzmann R, Hillenkamp J, Curcio CA, et al. (2020). Autofluorescent Granules of the Human Retinal Pigment Epithelium: Phenotypes, Intracellular Distribution, and Age-Related Topography. Invest Ophthalmol Vis Sci, 61:35. 10.1167/iovs.61.5.35 PMC7405767 32433758

[72] Liu Z, Xu J, Ma Q, Zhang X, Yang Q, Wang L, et al. (2020). Glycolysis links reciprocal activation of myeloid cells and endothelial cells in the retinal angiogenic niche. Sci Transl Med, 12:eaay1371. 32759274 10.1126/scitranslmed.aay1371 PMC7751280

[73] Saada J, McAuley RJ, Marcatti M, Tang TZ, Motamedi M, Szczesny B (2022). Oxidative stress induces Z-DNA-binding protein 1-dependent activation of microglia via mtDNA released from retinal pigment epithelial cells. J Biol Chem, 298:101523. 34953858 10.1016/j.jbc.2021.101523 PMC8753185

[74] Wang SK, Xue Y, Cepko CL (2020). Microglia modulation by TGF-beta1 protects cones in mouse models of retinal degeneration. J Clin Invest, 130:4360-4369. 32352930 10.1172/JCI136160 PMC7410072

[75] Fudalej E, Justyniarska M, Kasarello K, Dziedziak J, Szaflik JP, Cudnoch-Jedrzejewska A (2021). Neuroprotective Factors of the Retina and Their Role in Promoting Survival of Retinal Ganglion Cells: A Review. Ophthalmic Res, 64:345-355. 33454713 10.1159/000514441

[76] Kimura A NK, Guo X, Harada C, Harada T. (2016). Neuroprotection, Growth Factors and BDNF-TrkB Signalling in Retinal Degeneration. Int J Mol Sci, 17:1584. 27657046 10.3390/ijms17091584 PMC5037849

[77] Forouzanfar F, Shojapour M, Aghili ZS, Asgharzade S (2020). Growth Factors as Tools in Photoreceptor Cell Regeneration and Vision Recovery. Curr Drug Targets, 21:573-581. 31755378 10.2174/1389450120666191121103831

[78] Chakravarti S, Li R, Wen R, Banzon T, Maminishkis A, Miller SS (2011). CNTF Mediates Neurotrophic Factor Secretion and Fluid Absorption in Human Retinal Pigment Epithelium. PLoS ONE, 6:e23148. 21912637 10.1371/journal.pone.0023148 PMC3166283

[79] Li S, Sato K, Gordon WC, Sendtner M, Bazan NG, Jin M (2018). Ciliary neurotrophic factor (CNTF) protects retinal cone and rod photoreceptors by suppressing excessive formation of the visual pigments. J Biol Chem, 293:15256-15268. 30115683 10.1074/jbc.RA118.004008 PMC6166737

[80] Boyce M, Xin Y, Chowdhury O, Shang P, Liu H, Koontz V, et al. (2022). Microglia-Neutrophil Interactions Drive Dry AMD-like Pathology in a Mouse Model. Cells, 11:3535. 36428965 10.3390/cells11223535 PMC9688699

[81] Silverman SM, Wong WT (2018). Microglia in the Retina: Roles in Development, Maturity, and Disease. Annu Rev Vis Sci, 4:45-77. 29852094 10.1146/annurev-vision-091517-034425

[82] Nebel C AA, Rashid K, Langmann T. (2017). Activated microglia trigger inflammasome activation and lysosomal destabilization in human RPE cells. Biochem Biophys Res Commun, 484:681-686. 28159556 10.1016/j.bbrc.2017.01.176

[83] Gupta N BK, Milam AH. (2003). Activated microglia in human retinitis pigmentosa, late-onset retinal degeneration, and age-related macular degeneration. Exp Eye Res, 76:463-471. 12634111 10.1016/s0014-4835(02)00332-9

[84] Blasiak J, Sobczuk P, Pawlowska E, Kaarniranta K (2022). Interplay between aging and other factors of the pathogenesis of age-related macular degeneration. Ageing Res Rev, 81:101735. 36113764 10.1016/j.arr.2022.101735

[85] Jo DH, Yun JH, Cho CS, Kim JH, Kim JH, Cho CH (2019). Interaction between microglia and retinal pigment epithelial cells determines the integrity of outer blood-retinal barrier in diabetic retinopathy. Glia, 67:321-331. 30444022 10.1002/glia.23542

[86] Jeon CJ, Strettoi E, Masland RH (1998). The major cell populations of the mouse retina. J Neurosci, 18:8936-8946. 9786999 10.1523/JNEUROSCI.18-21-08936.1998 PMC6793518

[87] Lu YB, Franze K, Seifert G, Steinhäuser C, Kirchhoff F, Wolburg H, et al. (2006). Viscoelastic properties of individual glial cells and neurons in the CNS. Proc Natl Acad Sci U S A, 103:17759-17764. 17093050 10.1073/pnas.0606150103 PMC1693820

[88] Zahs KR CP (2006). Gap junctional coupling and connexin immunoreactivity in rabbit retinal glia. Vis Neurosci, 23:1-10. 16597346 10.1017/S0952523806231018

[89] Hippert C, Graca AB, Barber AC, West EL, Smith AJ, Ali RR, et al. (2015). Muller glia activation in response to inherited retinal degeneration is highly varied and disease-specific. PLoS One, 10:e0120415. 25793273 10.1371/journal.pone.0120415 PMC4368159

[90] Bringmann A, Wiedemann P (2012). Muller glial cells in retinal disease. Ophthalmologica, 227:1-19. 10.1159/000328979 21921569

[91] Bringmann A, Iandiev I, Pannicke T, Wurm A, Hollborn M, Wiedemann P, et al. (2009). Cellular signaling and factors involved in Müller cell gliosis: neuroprotective and detrimental effects. Prog Retin Eye Res, 28:423-451. 19660572 10.1016/j.preteyeres.2009.07.001

[92] Reichenbach A, Bringmann A (2013). New functions of Müller cells. Glia, 61:651-678. 23440929 10.1002/glia.22477

[93] Chu Y, Alder VA, Humphrey MF, Constable IJ (1999). Localization of IgG in the normal and dystrophic rat retina after laser lesions. Aust N Z J Ophthalmol, 27:117-125. 10379710 10.1046/j.1440-1606.1999.00164.x

[94] Li J, Yu S, Lu X, Cui K, Tang X, Xu Y, et al. (2021). The phase changes of M1/M2 phenotype of microglia/macrophage following oxygen-induced retinopathy in mice. Inflamm Res, 70:183-192. 33386422 10.1007/s00011-020-01427-w

[95] Wu KH, Madigan MC, Billson FA, Penfold PL (2003). Differential expression of GFAP in early v late AMD: a quantitative analysis. Br J Ophthalmol, 87:1159-1166. 12928288 10.1136/bjo.87.9.1159 PMC1771844

[96] Crabb JW (2014). The proteomics of drusen. Cold Spring Harb Perspect Med, 4:a017194. 24799364 10.1101/cshperspect.a017194 PMC4066642

[97] Graca AB, Hippert C, Pearson RA (2018). Muller Glia Reactivity and Development of Gliosis in Response to Pathological Conditions. Adv Exp Med Biol, 1074:303-308. 29721957 10.1007/978-3-319-75402-4_37

[98] Jünemann AG, Rejdak R, Huchzermeyer C, Maciejewski R, Grieb P, Kruse FE, et al. (2015). Elevated vitreous body glial fibrillary acidic protein in retinal diseases. Graefes Arch Clin Exp Ophthalmol, 253:2181-2186. 26279003 10.1007/s00417-015-3127-7 PMC4653239

[99] Yasuhara T, Shingo T, Date I (2004). The potential role of vascular endothelial growth factor in the central nervous system. Rev Neurosci, 15:293-307. 15526553 10.1515/revneuro.2004.15.4.293

[100] Bringmann A, Francke M, Pannicke T, Biedermann B, Kodal H, Faude F, et al. (2000). Role of glial K(+) channels in ontogeny and gliosis: a hypothesis based upon studies on Müller cells. Glia, 29:35-44. 10594921 10.1002/(sici)1098-1136(20000101)29:1<35::aid-glia4>3.0.co;2-a

[101] Karlstetter M, Ebert S, Langmann T (2010). Microglia in the healthy and degenerating retina: insights from novel mouse models. Immunobiology, 215:685-691. 20573418 10.1016/j.imbio.2010.05.010

[102] Wang M WX, Zhao L, Ma W, Rodriguez IR, Fariss RN, Wong WT. (2014). Macroglia-microglia interactions via TSPO signaling regulates microglial activation in the mouse retina. J Neurosci, 34:3793-3806. 24599476 10.1523/JNEUROSCI.3153-13.2014 PMC3942591

[103] Perry VH, Teeling J (2013). Microglia and macrophages of the central nervous system: the contribution of microglia priming and systemic inflammation to chronic neurodegeneration. Semin Immunopathol, 35:601-612. 23732506 10.1007/s00281-013-0382-8 PMC3742955

[104] Au NPB, Ma CHE (2022). Neuroinflammation, Microglia and Implications for Retinal Ganglion Cell Survival and Axon Regeneration in Traumatic Optic Neuropathy. Front Immunol, 13:860070. 35309305 10.3389/fimmu.2022.860070 PMC8931466

[105] Xue B, Xie Y, Xue Y, Hu N, Zhang G, Guan H, et al. (2016). Involvement of P2X(7) receptors in retinal ganglion cell apoptosis induced by activated Müller cells. Exp Eye Res, 153:42-50. 27720859 10.1016/j.exer.2016.10.005

[106] Xing L, Yang T, Cui S, Chen G (2019). Connexin Hemichannels in Astrocytes: Role in CNS Disorders. Front Mol Neurosci, 12:23. 30787868 10.3389/fnmol.2019.00023 PMC6372977

[107] Hu X, Zhao GL, Xu MX, Zhou H, Li F, Miao Y, et al. (2021). Interplay between Muller cells and microglia aggravates retinal inflammatory response in experimental glaucoma. J Neuroinflammation, 18:303. 34952606 10.1186/s12974-021-02366-x PMC8705189

[108] Platania CBM, Drago F, Bucolo C (2022). The P2X7 receptor as a new pharmacological target for retinal diseases. Biochem Pharmacol, 198:114942. 35134386 10.1016/j.bcp.2022.114942

[109] Xia J, Lim JC, Lu W, Beckel JM, Macarak EJ, Laties AM, et al. (2012). Neurons respond directly to mechanical deformation with pannexin-mediated ATP release and autostimulation of P2X7 receptors. J Physiol, 590:2285-2304. 22411013 10.1113/jphysiol.2012.227983 PMC3424753

[110] Campagno KE, Lu W, Jassim AH, Albalawi F, Cenaj A, Tso HY, et al. (2021). Rapid morphologic changes to microglial cells and upregulation of mixed microglial activation state markers induced by P2X7 receptor stimulation and increased intraocular pressure. J Neuroinflammation, 18:217. 34544431 10.1186/s12974-021-02251-7 PMC8454080

[111] Romano GL, Amato R, Lazzara F, Porciatti V, Chou TH, Drago F, et al. (2020). P2X7 receptor antagonism preserves retinal ganglion cells in glaucomatous mice. Biochem Pharmacol, 180:114199. 32798466 10.1016/j.bcp.2020.114199

[112] Clapp C, Diaz-Lezama N, Adan-Castro E, Ramirez-Hernandez G, Moreno-Carranza B, Sarti AC, et al. (2019). Pharmacological blockade of the P2X7 receptor reverses retinal damage in a rat model of type 1 diabetes. Acta Diabetol, 56:1031-1036. 30982154 10.1007/s00592-019-01343-4

[113] Lee KS, Lin S, Copland DA, Dick AD, Liu J (2021). Cellular senescence in the aging retina and developments of senotherapies for age-related macular degeneration. J Neuroinflammation, 18:32. 33482879 10.1186/s12974-021-02088-0 PMC7821689

[114] Li L, Eter N, Heiduschka P (2015). The microglia in healthy and diseased retina. Exp Eye Res, 136:116-130. 25952657 10.1016/j.exer.2015.04.020

[115] Bringmann A, Pannicke T, Grosche J, Francke M, Wiedemann P, Skatchkov S, et al. (2006). Müller cells in the healthy and diseased retina. Progress in Retinal and Eye Research, 25:397-424. 16839797 10.1016/j.preteyeres.2006.05.003

[116] Dick AD (2017). Doyne lecture 2016: intraocular health and the many faces of inflammation. Eye (Lond), 31:87-96. 27636226 10.1038/eye.2016.177 PMC5233925

[117] Wang Y, Wang VM, Chan CC (2011). The role of anti-inflammatory agents in age-related macular degeneration (AMD) treatment. Eye (Lond), 25:127-139. 21183941 10.1038/eye.2010.196 PMC3044916

[118] Ammar MJ, Hsu J, Chiang A, Ho AC, Regillo CD (2020). Age-related macular degeneration therapy: a review. Curr Opin Ophthalmol, 31:215-221. 32205470 10.1097/ICU.0000000000000657

[119] Al-Zamil WM, Yassin SA (2017). Recent developments in age-related macular degeneration: a review. Clin Interv Aging, 12:1313-1330. 28860733 10.2147/CIA.S143508 PMC5573066

[120] Homsi S, Federico F, Croci N, Palmier B, Plotkine M, Marchand-Leroux C, et al. (2009). Minocycline effects on cerebral edema: relations with inflammatory and oxidative stress markers following traumatic brain injury in mice. Brain Res, 1291:122-132. 19631631 10.1016/j.brainres.2009.07.031

[121] Scholz R, Sobotka M, Caramoy A, Stempfl T, Moehle C, Langmann T (2015). Minocycline counter-regulates pro-inflammatory microglia responses in the retina and protects from degeneration. J Neuroinflammation, 12:209. 26576678 10.1186/s12974-015-0431-4 PMC4650866

[122] Racic T, Chang A, Fernando N, Brandli A, Natoli R, Penfold P, et al. (2021). Anti-inflammatory and neuroprotective properties of the corticosteroid fludrocortisone in retinal degeneration. Exp Eye Res, 212:108765. 34509498 10.1016/j.exer.2021.108765

[123] Dot C, Behar-Cohen F, BenEzra D, Doat M, Jonet L, May F, et al. (2007). Influence of triamcinolone intravitreal injection on retinochoroidal healing processes. Exp Eye Res, 84:1081-1089. 17408616 10.1016/j.exer.2007.01.024

[124] Dessalces E, Bocquet B, Bourien J, Zanlonghi X, Verdet R, Meunier I, et al. (2013). Early-onset foveal involvement in retinitis punctata albescens with mutations in RLBP1. JAMA Ophthalmol, 131:1314-1323. 23929416 10.1001/jamaophthalmol.2013.4476

[125] Shen W, Lee S-R, Araujo J, Chung SH, Zhu L, Gillies MC (2014). Effect of glucocorticoids on neuronal and vascular pathology in a transgenic model of selective Müller cell ablation. Glia, 62:1110-1124. 24687761 10.1002/glia.22666

[126] Shen W, Fruttiger M, Zhu L, Chung SH, Barnett NL, Kirk JK, et al. (2012). Conditional Mullercell ablation causes independent neuronal and vascular pathologies in a novel transgenic model. J Neurosci, 32:15715-15727. 23136411 10.1523/JNEUROSCI.2841-12.2012 PMC4014009

[127] Xu J, Tu Y, Wang Y, Xu X, Sun X, Xie L, et al. (2020). Prodrug of epigallocatechin-3-gallate alleviates choroidal neovascularization via down-regulating HIF-1alpha/VEGF/VEGFR2 pathway and M1 type macrophage/microglia polarization. Biomed Pharmacother, 121:109606. 31743875 10.1016/j.biopha.2019.109606

[128] Ma W, Zhao L, Fontainhas AM, Fariss RN, Wong WT (2009). Microglia in the mouse retina alter the structure and function of retinal pigmented epithelial cells: a potential cellular interaction relevant to AMD. PLoS One, 4:e7945. 19936204 10.1371/journal.pone.0007945 PMC2775955

[129] Lancon A, Frazzi R, Latruffe N (2016). Anti-Oxidant, Anti-Inflammatory and Anti-Angiogenic Properties of Resveratrol in Ocular Diseases. Molecules, 21:304. 26950104 10.3390/molecules21030304 PMC6272926

[130] Yang PM, Cheng KC, Yuan SH, Wung BS (2020). Carbon monoxide-releasing molecules protect against blue light exposure and inflammation in retinal pigment epithelial cells. Int J Mol Med, 46:1096-1106. 32582966 10.3892/ijmm.2020.4656 PMC7387094

[131] Radomska-Lesniewska DM, Osiecka-Iwan A, Hyc A, Gozdz A, Dabrowska AM, Skopinski P (2019). Therapeutic potential of curcumin in eye diseases. Cent Eur J Immunol, 44:181-189. 31530988 10.5114/ceji.2019.87070 PMC6745545

[132] Zhu W, Wu Y, Meng YF, Wang JY, Xu M, Tao JJ, et al. (2015). Effect of curcumin on aging retinal pigment epithelial cells. Drug Des Devel Ther, 9:5337-5344. 10.2147/DDDT.S84979 PMC4590412 26445530

[133] Kim BJ, Mastellos DC, Li Y, Dunaief JL, Lambris JD (2021). Targeting complement components C3 and C5 for the retina: Key concepts and lingering questions. Prog Retin Eye Res, 83:100936. 33321207 10.1016/j.preteyeres.2020.100936 PMC8197769

[134] Anderson DH, Radeke MJ, Gallo NB, Chapin EA, Johnson PT, Curletti CR, et al. (2010). The pivotal role of the complement system in aging and age-related macular degeneration: hypothesis re-visited. Prog Retin Eye Res, 29:95-112. 19961953 10.1016/j.preteyeres.2009.11.003 PMC3641842

[135] Fett AL, Hermann MM, Muether PS, Kirchhof B, Fauser S (2012). Immunohistochemical localization of complement regulatory proteins in the human retina. Histol Histopathol, 27:357-364. 22237713 10.14670/HH-27.357

[136] Irfan M, Kim JH, Druzinsky RE, Ravindran S, Chung S (2022). Complement C5aR/LPS-induced BDNF and NGF modulation in human dental pulp stem cells. Sci Rep, 12:2042. 35132159 10.1038/s41598-022-06110-0 PMC8821590

[137] Kim SJ, Kim J, Lee J, Cho SY, Kang HJ, Kim KY, et al. (2013). Intravitreal human complement factor H in a rat model of laser-induced choroidal neovascularisation. Br J Ophthalmol, 97:367-370. 23258212 10.1136/bjophthalmol-2012-302307

[138] Bora NS, Jha P, Lyzogubov VV, Kaliappan S, Liu J, Tytarenko RG, et al. (2010). Recombinant membrane-targeted form of CD59 inhibits the growth of choroidal neovascular complex in mice. J Biol Chem, 285:33826-33833. 20736175 10.1074/jbc.M110.153130 PMC2962482

[139] Lundh von Leithner P, Kam JH, Bainbridge J, Catchpole I, Gough G, Coffey P, et al. (2009). Complement factor h is critical in the maintenance of retinal perfusion. Am J Pathol, 175:412-421. 19541934 10.2353/ajpath.2009.080927 PMC2708826

[140] Natoli R, Fernando N, Jiao H, Racic T, Madigan M, Barnett NL, et al. (2017). Retinal Macrophages Synthesize C3 and Activate Complement in AMD and in Models of Focal Retinal Degeneration. Invest Ophthalmol Vis Sci, 58:2977-2990. 28605809 10.1167/iovs.17-21672

[141] Mastellos DC, Yancopoulou D, Kokkinos P, Huber-Lang M, Hajishengallis G, Biglarnia AR, et al. (2015). Compstatin: a C3-targeted complement inhibitor reaching its prime for bedside intervention. European Journal of Clinical Investigation, 45:423-440. 25678219 10.1111/eci.12419 PMC4380746

[142] Kassa E, Ciulla TA, Hussain RM, Dugel PU (2019). Complement inhibition as a therapeutic strategy in retinal disorders. Expert Opin Biol Ther, 19:335-342. 30686077 10.1080/14712598.2019.1575358

[143] Park YG, Park YS, Kim IB (2021). Complement System and Potential Therapeutics in Age-Related Macular Degeneration. Int J Mol Sci, 22:6851. 34202223 10.3390/ijms22136851 PMC8269056

[144] Cammalleri M, Dal Monte M, Locri F, Lardner E, Kvanta A, Rusciano D, et al. (2017). Efficacy of a Fatty Acids Dietary Supplement in a Polyethylene Glycol-Induced Mouse Model of Retinal Degeneration. Nutrients, 9:1079. 28961167 10.3390/nu9101079 PMC5691696

[145] Jia H, Li T, Sun J, Gong Y, Liu H, Wang H, et al. (2023). A Novel Bispecific Fusion Protein Targeting C3b/C4b and VEGF in Patients With nAMD: A Randomized, Open-Label, Phase 1b Study. Am J Ophthalmol, 248:8-15. 36410472 10.1016/j.ajo.2022.11.016

[146] Clark SJ, Bishop PN, Day AJ (2010). Complement factor H and age-related macular degeneration: the role of glycosaminoglycan recognition in disease pathology. Biochem Soc Trans, 38:1342-1348. 20863311 10.1042/BST0381342

[147] Blom AM, Kask L, Ramesh B, Hillarp A (2003). Effects of zinc on factor I cofactor activity of C4b-binding protein and factor H. Archives of Biochemistry and Biophysics, 418:108-118. 14522582 10.1016/j.abb.2003.08.018

[148] Borras C, Delaunay K, Slaoui Y, Abache T, Jorieux S, Naud MC, et al. (2020). Mechanisms of FH Protection Against Neovascular AMD. Front Immunol, 11:443. 32318056 10.3389/fimmu.2020.00443 PMC7146894

[149] Park DW, Lee YG, Jeong YJ, Jeon H, Kang SC (2021). Preventive Effects against Retinal Degeneration by Centella asiatica Extract (CA-HE50) and Asiaticoside through Apoptosis Suppression by the Nrf2/HO-1 Signaling Pathway. Antioxidants (Basel), 10:613. 33923585 10.3390/antiox10040613 PMC8072678

[150] Lim W-C, Kim H, Kim Y-J, Park S-H, Song J-H, Lee KH, et al. (2017). Delphinidin inhibits BDNF-induced migration and invasion in SKOV3 ovarian cancer cells. Bioorganic & Medicinal Chemistry Letters, 27:5337-5343. 29122484 10.1016/j.bmcl.2017.09.024

[151] Ni T, Yang W, Xing Y (2019). Protective effects of delphinidin against H2O2-induced oxidative injuries in human retinal pigment epithelial cells. Bioscience Reports, 39:BSR20190689. 31345961 10.1042/BSR20190689 PMC6695502

[152] Zhu YX, Yao J, Liu C, Hu HT, Li XM, Ge HM, et al. (2018). Long non-coding RNA MEG3 silencing protects against light-induced retinal degeneration. Biochem Biophys Res Commun, 496:1236-1242. 29409883 10.1016/j.bbrc.2018.01.177

[153] Wu H, Xu J, Du X, Cui J, Zhang T, Chen Y (2020). Shihu Yeguang Pill protects against bright light-induced photoreceptor degeneration in part through suppressing photoreceptor apoptosis. Biomed Pharmacother, 126:110050. 32135462 10.1016/j.biopha.2020.110050

[154] Gong X, Draper CS, Allison GS, Marisiddaiah R, Rubin LP (2017). Effects of the Macular Carotenoid Lutein in Human Retinal Pigment Epithelial Cells. Antioxidants (Basel), 6:100. 29207534 10.3390/antiox6040100 PMC5745510

[155] Lan L, Wang S, Duan S, Zhou X, Li Y, Yang S (2022). Cordyceps militaris Carotenoids Protect Human Retinal Endothelial Cells against the Oxidative Injury and Apoptosis Resulting from H2O2. Evidence-Based Complementary and Alternative Medicine, 2022:1-12. 10.1155/2022/1259093 PMC9546680 36212977

[156] Bhattacharyya J, Sharma KK (2001). Conformational specificity of mini-alphaA-crystallin as a molecular chaperone. J Pept Res, 57:428-434. 11350603 10.1034/j.1399-3011.2001.00871.x

[157] Chen Q, Lin H, Li S, Deng X, Zhang J (2023). Mini-alphaA Upregulates the miR-155-5p Target Gene CDK2 and Plays an Antiapoptotic Role in Retinal Pigment Epithelial Cells during Oxidative Stress. J Ophthalmol, 2023:6713094. 36824443 10.1155/2023/6713094 PMC9943629

[158] Chuang CJ, Wang M, Yeh JH, Chen TC, Tsou SC, Lee YJ, et al. (2021). The Protective Effects of alpha-Mangostin Attenuate Sodium Iodate-Induced Cytotoxicity and Oxidative Injury via Mediating SIRT-3 Inactivation via the PI3K/AKT/PGC-1alpha Pathway. Antioxidants (Basel), 10:1870. 34942973 10.3390/antiox10121870 PMC8698330

[159] Garber K (2015). RIKEN suspends first clinical trial involving induced pluripotent stem cells. Nat Biotechnol, 33:890-891. 26348942 10.1038/nbt0915-890

[160] Falsini B, Bisti S (2012). Embryonic stem-cell-derived retinal pigment epithelial cells for macular degeneration. Lancet, 379:2050; author reply 2050-2051. 10.1016/S0140-6736(12)60890-3 22656882

[161] da Cruz L, Fynes K, Georgiadis O, Kerby J, Luo YH, Ahmado A, et al. (2018). Phase 1 clinical study of an embryonic stem cell-derived retinal pigment epithelium patch in age-related macular degeneration. Nat Biotechnol, 36:328-337. 29553577 10.1038/nbt.4114

[162] Petrus-Reurer S, Kumar P, Padrell Sanchez S, Aronsson M, Andre H, Bartuma H, et al. (2020). Preclinical safety studies of human embryonic stem cell-derived retinal pigment epithelial cells for the treatment of age-related macular degeneration. Stem Cells Transl Med, 9:936-953. 32319201 10.1002/sctm.19-0396 PMC7381808

[163] Wang Z, Gao F, Zhang M, Zheng Y, Zhang F, Xu L, et al. (2020). Intravitreal Injection of Human Retinal Progenitor Cells for Treatment of Retinal Degeneration. Med Sci Monit, 26:e921184. 32221273 10.12659/MSM.921184 PMC7139196

[164] Zhu D, Xie M, Gademann F, Cao J, Wang P, Guo Y, et al. (2020). Protective effects of human iPS-derived retinal pigmented epithelial cells on retinal degenerative disease. Stem Cell Res Ther, 11:98. 32131893 10.1186/s13287-020-01608-8 PMC7055119

[165] Sharma A, Jaganathan BG (2021). Stem Cell Therapy for Retinal Degeneration: The Evidence to Date. Biologics, 15:299-306. 34349498 10.2147/BTT.S290331 PMC8327474

[166] Wen YT, Ho YC, Lee YC, Ding DC, Liu PK, Tsai RK (2021). The Benefits and Hazards of Intravitreal Mesenchymal Stem Cell (MSC) Based-Therapies in the Experimental Ischemic Optic Neuropathy. Int J Mol Sci, 22:2117. 33672743 10.3390/ijms22042117 PMC7924624

[167] Mehta H, Tufail A, Daien V, Lee AY, Nguyen V, Ozturk M, et al. (2018). Real-world outcomes in patients with neovascular age-related macular degeneration treated with intravitreal vascular endothelial growth factor inhibitors. Prog Retin Eye Res, 65:127-146. 29305324 10.1016/j.preteyeres.2017.12.002

[168] Group CR, Martin DF, Maguire MG, Ying GS, Grunwald JE, Fine SL, et al. (2011). Ranibizumab and bevacizumab for neovascular age-related macular degeneration. N Engl J Med, 364:1897-1908. 21526923 10.1056/NEJMoa1102673 PMC3157322

[169] Bhisitkul RB, Desai SJ, Boyer DS, Sadda SR, Zhang K (2016). Fellow Eye Comparisons for 7-Year Outcomes in Ranibizumab-Treated AMD Subjects from ANCHOR, MARINA, and HORIZON (SEVEN-UP Study). Ophthalmology, 123:1269-1277. 26996339 10.1016/j.ophtha.2016.01.033

[170] Ricci F, Bandello F, Navarra P, Staurenghi G, Stumpp M, Zarbin M (2020). Neovascular Age-Related Macular Degeneration: Therapeutic Management and New-Upcoming Approaches. Int J Mol Sci, 21:8242. 33153227 10.3390/ijms21218242 PMC7662479

[171] Zhou B, Wang B (2006). Pegaptanib for the treatment of age-related macular degeneration. Exp Eye Res, 83:615-619. 16678158 10.1016/j.exer.2006.02.010

[172] Garcia J, Hurwitz HI, Sandler AB, Miles D, Coleman RL, Deurloo R, et al. (2020). Bevacizumab (Avastin(R)) in cancer treatment: A review of 15 years of clinical experience and future outlook. Cancer Treat Rev, 86:102017. 32335505 10.1016/j.ctrv.2020.102017

[173] Caceres-Del-Carpio J, Moustafa MT, Toledo-Corral J, Hamid MA, Atilano SR, Schneider K, et al. (2020). In vitro response and gene expression of human retinal Muller cells treated with different anti-VEGF drugs. Exp Eye Res, 191:107903. 31904361 10.1016/j.exer.2019.107903 PMC7058176

[174] Tan CS, Ngo WK, Chay IW, Ting DS, Sadda SR (2022). Neovascular Age-Related Macular Degeneration (nAMD): A Review of Emerging Treatment Options. Clin Ophthalmol, 16:917-933. 35368240 10.2147/OPTH.S231913 PMC8965014

[175] Damkondwar DR, Srinivasan R, Raman R, Kulothungan V, Sharma T (2022). Morphological and functional retinal changes in neovascular age-related macular degeneration treated with intravitreal bevacizumab. Indian J Ophthalmol, 70:4376-4382. 36453348 10.4103/ijo.IJO_1184_22 PMC9940573

[176] Ng DSC, Fung NSK, Yip FLT, Lai TYY (2020). Ranibizumab for myopic choroidal neovascularization. Expert Opin Biol Ther, 20:1385-1393. 33003962 10.1080/14712598.2021.1830969

[177] Papadopoulos N, Martin J, Ruan Q, Rafique A, Rosconi MP, Shi E, et al. (2012). Binding and neutralization of vascular endothelial growth factor (VEGF) and related ligands by VEGF Trap, ranibizumab and bevacizumab. Angiogenesis, 15:171-185. 22302382 10.1007/s10456-011-9249-6 PMC3338918

[178] Ho M, Woo DC, Chan VC, Young AL, Brelen ME (2016). Treatment of polypoidal choroidal vasculopathy by photodynamic therapy, aflibercept and dexamethasone triple therapy. Sci Rep, 6:36870. 27848983 10.1038/srep36870 PMC5111116

[179] Nguyen QD, De Falco S, Behar-Cohen F, Lam WC, Li X, Reichhart N, et al. (2018). Placental growth factor and its potential role in diabetic retinopathy and other ocular neovascular diseases. Acta Ophthalmol, 96:e1-e9. 27874278 10.1111/aos.13325 PMC5811779

[180] Lazzara F, Fidilio A, Platania CBM, Giurdanella G, Salomone S, Leggio GM, et al. (2019). Aflibercept regulates retinal inflammation elicited by high glucose via the PlGF/ERK pathway. Biochem Pharmacol, 168:341-351. 31351870 10.1016/j.bcp.2019.07.021

[181] Rojo Arias JE, Englmaier VE, Jaszai J (2022). VEGF-Trap Modulates Retinal Inflammation in the Murine Oxygen-Induced Retinopathy (OIR) Model. Biomedicines, 10:201. 35203414 10.3390/biomedicines10020201 PMC8869660

[182] M S (2022). Faricimab: First Approval. Drugs, 82(7):825-830. 35474059 10.1007/s40265-022-01713-3

[183] Schaefer W, Regula JT, Bahner M, Schanzer J, Croasdale R, Durr H, et al. (2011). Immunoglobulin domain crossover as a generic approach for the production of bispecific IgG antibodies. Proc Natl Acad Sci U S A, 108:11187-11192. 21690412 10.1073/pnas.1019002108 PMC3131342

[184] Heier JS, Khanani AM, Quezada Ruiz C, Basu K, Ferrone PJ, Brittain C, et al. (2022). Efficacy, durability, and safety of intravitreal faricimab up to every 16 weeks for neovascular age-related macular degeneration (TENAYA and LUCERNE): two randomised, double-masked, phase 3, non-inferiority trials. Lancet, 399:729-740. 35085502 10.1016/S0140-6736(22)00010-1

[185] Sahni J, Patel SS, Dugel PU, Khanani AM, Jhaveri CD, Wykoff CC, et al. (2019). Simultaneous Inhibition of Angiopoietin-2 and Vascular Endothelial Growth Factor-A with Faricimab in Diabetic Macular Edema: BOULEVARD Phase 2 Randomized Trial. Ophthalmology, 126:1155-1170. 30905643 10.1016/j.ophtha.2019.03.023

[186] Tadayoni R, Sararols L, Weissgerber G, Verma R, Clemens A, Holz FG (2021). Brolucizumab: A Newly Developed Anti-VEGF Molecule for the Treatment of Neovascular Age-Related Macular Degeneration. Ophthalmologica, 244:93-101. 33197916 10.1159/000513048

[187] Finger RP, Dennis N, Freitas R, Quenechdu A, Clemens A, Karcher H, et al. (2022). Comparative Efficacy of Brolucizumab in the Treatment of Neovascular Age-Related Macular Degeneration: A Systematic Literature Review and Network Meta-Analysis. Adv Ther, 39:3425-3448. 35678996 10.1007/s12325-022-02193-3 PMC9309118

[188] Holz FG, Dugel PU, Weissgerber G, Hamilton R, Silva R, Bandello F, et al. (2016). Single-Chain Antibody Fragment VEGF Inhibitor RTH258 for Neovascular Age-Related Macular Degeneration: A Randomized Controlled Study. Ophthalmology, 123:1080-1089. 26906165 10.1016/j.ophtha.2015.12.030

[189] Avery RL CA, Steinle NC, Dhoot DS, Pieramici DJ, See R, Couvillion S, Nasir MA, Rabena MD, Maia M, Van Everen S, Le K, Hanley WD. (2017). Systemic pharmacokinetics and ohaemacodynamics of intravitreal aflibercept,bevacizumab and ranibizumab. Retina, 37:1847-1858. 28106709 10.1097/IAE.0000000000001493 PMC5642319

[190] Wang Y, Fang Q, Zhang C, Chen Y, Gou T, Cai Q, et al. (2021). Multimodal imaging and electroretinography highlights the role of VEGF in the laser-induced subretinal fibrosis of monkey. Exp Eye Res, 203:108417. 33358768 10.1016/j.exer.2020.108417

[191] Zhang L, Si T, Fischer AJ, Letson A, Yuan S, Roberts CJ, et al. (2015). Coaxial Electrospray of Ranibizumab-Loaded Microparticles for Sustained Release of Anti-VEGF Therapies. PLoS One, 10:e0135608. 26273831 10.1371/journal.pone.0135608 PMC4537102

[192] Nussenblatt RB, Byrnes G, Sen HN, Yeh S, Faia L, Meyerle C, et al. (2010). A randomized pilot study of systemic immunosuppression in the treatment of age-related macular degeneration with choroidal neovascularization. Retina, 30:1579-1587. 20847709 10.1097/IAE.0b013e3181e7978e PMC3174007

[193] Ajish TP, Praveen VP, Nisha B, Kumar H (2014). Comparison of different glucocorticoid regimens in the management of classical congenital adrenal hyperplasia due to 21-hydroxylase deficiency. Indian J Endocrinol Metab, 18:815-820. 25364676 10.4103/2230-8210.141358 PMC4192987

[194] Lee A, Shirley M (2021). Ranibizumab: A Review in Retinopathy of Prematurity. Paediatr Drugs, 23:111-117. 33447937 10.1007/s40272-020-00433-z

